# Supplementary material for: Household structure and its association with sexual risk behaviours and sexual health outcomes: evidence from a British probability sample survey
Source: BMJ Open. 2018 Dec 4;8(12):e024255. doi: 10.1136/bmjopen-2018-024255 (PMC6286480; doi:10.1136/bmjopen-2018-024255)
Supplement: Author's manuscript [file bmjopen-2018-024255.draft_revisions.pdf]

BMJ Open is committed to open peer review. As part of this commitment we make the peer review history of every article we publish publicly available.

When an article is published we post the peer reviewers' comments and the authors' responses online. We also post the versions of the paper that were used during peer review. These are the versions that the peer review comments apply to.

The versions of the paper that follow are the versions that were submitted during the peer review process. They are not the versions of record or the final published versions. They should not be cited or distributed as the published version of this manuscript.

BMJ Open is an open access journal and the full, final, typeset and author-corrected version of record of the manuscript is available on our site with no access controls, subscription charges or pay-per-view fees (http://bmjopen.bmj.com).

If you have any questions on BMJ Open's open peer review process please email info.bmjopen@bmj.com

Household structure and its association with sexual behaviour and sexual health outcomes: Evidence from a British probability sample survey

Journal:	*BMJ Open*
Manuscript ID	bmjopen-2018-024255
Article Type:	Research
Date Submitted by the Author:	17-May-2018

Complete List of Authors:	Curtis, Tyrone; University College London Institute for Global Health, Centre for Population Research in Sexual Health and HIV<br>Field, Nigel; University College London Institute for Global Health, Centre for Molecular Epidemiology and Translational Research<br>Clifton, Soazig; University College London Institute for Global Health, Centre for Population Research in Sexual Health and HIV<br>Mercer, Catherine; University College London Institute for Global Health, Centre for Population Research in Sexual Health and HIV

Keywords:	GENITOURINARY MEDICINE, Epidemiology < INFECTIOUS DISEASES, SEXUAL MEDICINE, STATISTICS & RESEARCH METHODS

SCHOLARONE™ Manuscripts

Full title: Household structure and its association with sexual behaviour and sexual health outcomes: Evidence from a British probability sample survey

Short title: Household structure and sexual behaviour and sexual health

Authors:

Tyrone Curtis*, MSc[a]

Nigel Field, MB PhD[b]

Soazig Clifton, MSc[a]

Catherine H. Mercer, PhD[a]

[a]Centre for Population Research in Sexual Health and HIV, Institute for Global Health, University College London, London, United Kingdom

[b]Centre for Molecular Epidemiology and Translational Research, Institute for Global Health, University College London, London, United Kingdom

***Corresponding Author:** Tyrone Curtis, Centre for Population Research in Sexual Health and HIV, UCL, 3rd Floor, Mortimer Market Centre, off Capper Street, London WC1E 6JB; tyrone.curtis@ucl.ac.uk

Word count: 2959 words

For peer review only

ABSTRACT

Objectives: Information on household structure is often routinely collected in surveys to enable participant selection and develop statistical weights. A person's home-life context might also influence their sexual behaviour, with implications for their sexual healthcare needs; we investigated this hypothesis.

Methods: Britain's third National Survey of Sexual Attitudes and Lifestyles (Natsal-3), a probability sample survey undertaken in 2010-2012, interviewed 15,162 men and women aged 16-74 years in Britain (with 3,869 aged 16-24 years). We examined household structure by gender and age-group. We then focused on young people (aged 16-24 years), and used multivariable models to explore associations between household structure, sexual behaviours and sexual health outcomes (all past year), independent of confounders such as age, relationship status, employment, and area of residence.

Results: Young people were most likely to be living with parents (women 57.1% (95%CI 54.5%-59.6%) and men 68.7% (65.4%-71.8)), or non-relatives (women 10.5% (8.5%-12.9%) and men 12.6% (10.1%-15.6%)). A further 6.9% (5.9%-8.1%) of young women and 7.6% (6.2%-9.4%) of young men lived alone. Compared with young women living with parents (reference category), young women living alone and those living with non-relatives had a higher likelihood of reporting ≥2 sexual partners (age-adjusted odds ratio 1.54 (95%CI 1.03-2.31); 1.76 (1.03-3.00), respectively). Women living alone were also more likely to have had unsafe sex (2.04 (1.38-3.02)). Despite these differences in sexually transmitted infection risk, there was no difference in sexual healthcare-seeking behaviour. Associations between household structure and sexual behaviour were not observed in young men.

For peer review only

Conclusions: We found evidence of an association between household structure and sexual behaviour in young women (but not men), that was independent of age, relationship status and other demographic factors. Given changes in the living arrangements of young people in Britain, the potential role of household in sexual health research should be further investigated.

Total: 298 words

Article summary:

- This is the first study to investigate the association between household structure and sexual behaviour and sexual health among young people; a population group that bears a disproportionate burden of sexual ill-health.
- The study analysed data from a large, national probability sample survey of sexual health enabling us to examine a broad range of sexual behaviour and sexual health outcomes.
- We showed that household structure is independently associated with sexual behaviour among young women but not men.
- While the results can be considered as broadly representative of the British general population, the numbers of participants from ethnic minority groups were relatively small, and the sampling frame - private households - means that those living in institutions or homeless are excluded.
- Given ongoing changes in the living arrangements of young people in Britain, household structure should be considered in future sexual health research.

For peer review only

INTRODUCTION

Sexual health surveys such as Britain's National Survey of Sexual Attitudes and Lifestyles (Natsal) routinely collect information on respondents' household structure, that is, the composition of respondents' households based on their relationships to other household members. This information is used to derive demographic variables such as equivalised household income, and to calculate statistical weights so that the data are broadly representative of the population. While relationship or cohabitation status have often been shown to be associated with differences in sexual behaviour[1, 2] and sexual health outcomes,[3] household structure has rarely been investigated.

Young people in the UK are increasingly spending their early adulthood living with parents.[4] One reason for this may be the increased uptake of higher education, with twice as many students in 2014 living at home for the duration of their studies than in 1996.[5] Those students who do move away from home are increasingly likely to return to the parental home upon completion of their studies, if only for a brief period.[6] Traditionally, a key driver for leaving the parental home has been marriage or, more recently, to cohabit with a partner. However, young people are deferring the age at which they first move in with a partner, and so tend not to form new family units until later in their twenties and early thirties.[5] The living arrangements of young people are also heavily dependent on economic circumstances. The economic uncertainty caused by the financial crisis of 2007-08 and the recession that followed disproportionately affected young people, resulting in high levels of youth unemployment,[7] a factor known to increase the proportion of young people living at home with parents.[8] Restrictions in access to the welfare system and housing market have also made it more likely for young people outside of family households to be living in rented accommodation, often sharing with friends or other non-related adults.[9] Overall, more young people are living at home with their parents, and those who do leave home are more likely to be living for a time outside of a family household.

For peer review only

It is plausible that these changes might affect sexual behaviour, for example, due to changes in the opportunity to, and frequency with which, young people have sex, with implications for sexual health. However, to date, there has been no investigation of the relationship between household structure and sexual behaviour, or in turn, use of sexual healthcare. In this study, we therefore used nationally-representative data from Natsal-3 to explore the distribution of household structures for the British population aged 16-74 years, and then investigate the independent associations between household structure and the sexual health and sexual behaviour of young people aged 16-24 years, focusing on young people as the group that experiences the greatest social and sexual transitions[10] and among the highest burden of sexually transmitted infections (STIs).[11, 12]

METHODS

Participants and procedures

Full details of the methods of Natsal-3 have been reported elsewhere.[13, 14] Briefly, Natsal-3 was a survey of 15,162 men and women aged 16-74 years resident in Britain, undertaken from 2010 to 2012, and recruited using a multistage, clustered, stratified probability sample design. Included in the sample were 3,869 men and women aged 16-24 years (1,729 men). The Natsal-3 study was approved by the Oxfordshire Research Ethics Committee A (reference: 09/H0604/27). Participants provided oral informed consent for interviews.

Participants were interviewed in their own homes by professional interviewers using a combination of a face-to-face computer-administered personal interview (CAPI) and a computer-assisted self-interview (CASI). Most questions relating to general health and well-being were collected face-to-face.

More sensitive questions about participants' sexual behaviour and sexual health, including questions about STI testing and diagnoses, and sexual health clinic attendance, were asked via the CASI. Participants were routed into the CASI if they reported sex with an opposite-sex partner and/or reported any sexual experience with someone of the same sex (both since age 13), or if they refused

For peer review only

to answer this question. Participants reporting no previous sexual experience did not complete the CASI.

Recent sexual activity was defined as reporting one or more occasions of vaginal or oral or anal sex with a partner of the opposite sex, or oral or anal sex or genital contact with a partner of the same sex in the past 4 weeks. Unsafe sex was defined as reporting two or more partners of opposite or same sex with no condom use in the past year, or no condom use at first sex with one or more new partners of opposite or same sex in the past year. STI diagnosis in the past year was defined as reporting diagnosis with one of the following STIs in the past year: trichomoniasis, gonorrhoea, chlamydia, syphilis, non-specific or non-gonococcal urethritis (NSU/NGU), genital warts, or herpes.

After completing the CASI, participants were asked to provide information on up to 11 additional household members, including their age, sex, and relationship to the participant (partner, son or daughter (including step-, foster or -in-law), parent (including step-, foster or -in-law), brother/sister (including step-, half, foster or -in-law), grandchild, grandparent, other relative or non-relative). Information on other members of participants' households was used to devise six household structure categories (Table 1). In the criteria below, "children" and "grandchildren" refer to those of the participant, regardless of age and including step-, foster or -in-law.

Table 1 Household structure categories derived from Natsal questionnaire responses about other members of participants' households

Household structure category	Criteria
Lives with parents or other relatives	Lives with parents or other relatives (including siblings), but not with a partner or children. Household may include non-relatives.
Lives alone	No other household members.
Lives with non-relatives only	Does not live with parents, other relatives, a partner or children/grandchildren.
Lives with a partner, not with children/grandchildren	Lives with a partner but not with children or grandchildren. Household may include other members.

For peer review only

Lives with a partner and children/grandchildren	Lives with a partner and children or grandchildren. Household may include other members.
Lives with children/grandchildren, not with partner	Lives with children or grandchildren but does not live with a partner. Household may include other members.

Patient and Public Involvement

As an unfunded secondary analysis of Natsal-3 data, this particular paper did not entail Patient and Public Involvement. However, public engagement has been a key component of each phase of the (to date) three rounds of the Natsal study, including public consultation exercises to inform new topic areas, cognitive interviews to inform question wording, and interactive public engagement events (including via digital media) to disseminate findings.

Statistical analysis

All analyses were carried out using the complex survey functions of Stata (version 15.1) to incorporate the weighting, clustering and stratification of Natsal-3.[14]

We first calculated the distribution of household structure by gender and age-group for all participants aged 16-74 years (0.6% non-response). To examine how the reporting of sexual behaviour and sexual health outcomes by young people varied by household structure category, we used data for 1,717 women and 1,361 men aged 16-24 years who reported having had at least one sexual partner in their lifetime (hereon 'sexually-experienced young people'). We calculated descriptive statistics for each reported behaviour or outcome, and used binary logistic regression to calculate age-adjusted odds ratios (aAORs) to compare participants living with parents or other relatives with those living in other household structure categories. In multivariable models, we adjusted for variables in Natsal-3 considered as potentially confounding, including age, relationship status (in a steady relationship, not in a steady relationship), area of residence (rural, London, or other urban), and employment/student status (working, in full-time education, or not in work or education).

We used an alpha of 0.05 in all analyses.

For peer review only

RESULTS

Variations in household structure by gender and age-group

The majority of people aged 16-74 years in Britain in 2010 lived with a partner or a partner and children, with the proportions similar for men (62.6% (61.2%-64.0%)) and women (60.7% (59.6%-61.9%)) (Figure 1). However, there were differences by gender for those not living with partners. Women were more likely than men to be living with children but not with a partner (11.9% (11.1%-12.7%) vs 1.8% (1.4%-2.2%); p<0.001), and less likely than men to be living with their parents (11.2% (10.6%-11.8%) vs 16.0% (15.2%-17.0%); p<0.001). Older women were more likely to be living alone; 30.4% (27.6%-33.4%) of women aged 65 or older lived alone, compared with 19.3% (16.8%-22.1%) of men of the same age (p<0.001).

Among 16-24-year-olds, women were less likely to be living in their parents' home than men; 57.1% (54.5%-59.6%) of women lived with parents or other relatives, compared with 68.7% (65.4%-71.8%) of men of similar age (p<0.001). Women were more likely to be living in their own family households; 25.6% (23.6%-27.7%) lived with a partner and/or children, compared with 11.1% (9.5%-13.0%) of men (p<0.001). Young men and women were equally as likely to be living in non-family households; 7.6% (6.2%-9.4%) of men and 6.9% (5.9%-8.1%) of women lived alone, and 12.6% (10.1%-15.6%) of men and 10.5% (8.5%-12.9%) of women lived with non-relatives.

Variations in sexual risk behaviours and sexual health outcomes reported by sexually-experienced young people (aged 16-24 years) by household structure

Among sexually-experienced women, 50.8% lived with parents or other relatives, 7.4% lived alone, 10.6% lived with non-relatives only, 11.9% lived with a partner but not with children, 9.4% lived with a partner and children and 9.9% lived with children but not with a partner. Compared with young women living with parents or other relatives, those living alone, living with partners but not with children, or living with partners and children were more likely to report having had sex in the past 4 weeks (weighted prevalence 74.0% vs 83.7%, 97.7% and 90.2% respectively) [Table 2]. After adjusting for age, relationship status, area of residence and employment/student status, women living alone and women living with partners but not with children remained more likely to report

For peer review only - http://bmjopen.bmj.com/site/about/guidelines.xhtml

having had recent sex than their counterparts living with parents or other relatives (aAOR 1.84 (95% CI 1.11-3.04) and 3.32 (1.13-9.74) respectively). Similarly, women living alone (45.5% vs 40.7%; aAOR 1.54 (1.03-2.31)) or with non-relatives (47.5% vs 40.7%; aAOR 1.76 (1.03-3.00)) were more likely to report two or more partners in the past year. Women living alone were also more likely to have had unsafe sex in the past year (44.2% vs 32.7%; aAOR 2.04 (1.38-3.02)).

Table 2: Sexual behaviour and sexual health outcomes in sexually-experienced women aged 16-24 years

	Denominator (unweighted)	Denominator (weighted)	Prevalence (%)	OR	95% CI	aAOR*	95% CI
**SEXUAL BEHAVIOUR**							
**Any sexual intercourse in past 4 weeks**				p<0.001	"	p=0.02	"
Lives with parents or other relatives	849	472	74.0	1.00	"	1.00	"
Lives alone	176	70	83.7	1.80	(1.18-2.75)	1.84	(1.11-3.04)
Lives with non-relatives only	96	95	71.8	0.89	(0.54-1.47)	0.99	(0.51-1.90)
Lives with a partner, not with children/grandchildren	169	112	97.7	14.81	(5.37-40.86)	3.32	(1.13-9.74)
Lives with a partner and children/grandchildren	147	89	90.2	3.24	(1.76-5.99)	0.69	(0.35-1.38)
Lives with children/grandchildren, not with a partner	241	93	74.1	1.00	(0.68-1.47)	0.96	(0.60-1.53)
**Four or more occasions of sexual intercourse in past 4 weeks**				p<0.001	"	p=0.32	"
Lives with parents or other relatives	810	453	45.3	1.00	"	1.00	"
Lives alone	158	63	53.2	1.37	(0.94-2.00)	1.41	(0.89-2.23)
Lives with non-relatives only	90	90	44.5	0.97	(0.62-1.52)	1.21	(0.75-1.96)
Lives with a partner, not with children/grandchildren	151	101	67.2	2.47	(1.70-3.61)	1.03	(0.68-1.58)
Lives with a partner and children/grandchildren	137	83	60.9	1.88	(1.28-2.78)	0.80	(0.50-1.26)
Lives with children/grandchildren, not with a partner	224	86	40.5	0.82	(0.59-1.15)	0.81	(0.52-1.27)
**Two or more partners† in past year**				p<0.001	"	p<0.001	"
Lives with parents or other relatives	866	483	40.7	1.00	"	1.00	"
Lives alone	176	70	45.5	1.22	(0.84-1.77)	1.54	(1.03-2.31)
Lives with non-relatives only	99	99	47.5	1.32	(0.81-2.17)	1.76	(1.03-3.00)
Lives with a partner, not with children/grandchildren	169	112	9.9	0.16	(0.09-0.27)	0.28	(0.16-0.49)
Lives with a partner and children/grandchildren	148	90	6.5	0.10	(0.05-0.20)	0.17	(0.09-0.35)
Lives with children/grandchildren, not with a partner	245	94	37.3	0.87	(0.63-1.20)	1.00	(0.69-1.43)
**Had unsafe sex in past year§**				p<0.001	"	p<0.001	"
Lives with parents or other relatives	863	482	32.7	1.00	"	1.00	"
Lives alone	176	70	44.2	1.63	(1.12-2.38)	2.04	(1.38-3.02)
Lives with non-relatives only	98	96	26.6	0.75	(0.44-1.28)	1.07	(0.60-1.92)
Lives with a partner, not with children/grandchildren	169	112	12.1	0.28	(0.17-0.46)	0.48	(0.29-0.81)
Lives with a partner and children/grandchildren	149	90	14.9	0.36	(0.22-0.58)	0.62	(0.36-1.06)
Lives with children/grandchildren, not with a partner	246	94	35.8	1.15	(0.83-1.59)	1.41	(0.98-2.04)
**SEXUAL HEALTH**							
**Diagnosed with an STI in past year‡**				p=0.19	"	p=0.13	"
Lives with parents or other relatives	869	484	5.1	1.00	"	1.00	"

Lives alone	178	71	5.9	1.17	(0.53-2.57)	1.05	(0.41-2.68)
Lives with non-relatives only	101	101	4.8	0.93	(0.21-4.04)	0.94	(0.21-4.17)
Lives with a partner, not with children/grandchildren	170	113	3.3	0.64	(0.26-1.53)	0.51	(0.17-1.49)
Lives with a partner and children/grandchildren	149	90	0.8	0.16	(0.04-0.69)	0.12	(0.02-0.61)
Lives with children/grandchildren, not with a partner	244	94	4.3	0.83	(0.41-1.65)	0.67	(0.27-1.67)
**Tested for chlamydia in past year**				p<0.001	"	p<0.001	"
Lives with parents or other relatives	870	485	55.8	1.00	"	1.00	"
Lives alone	178	71	55.1	0.97	(0.67-1.41)	0.96	(0.65-1.43)
Lives with non-relatives only	101	101	57.1	1.05	(0.64-1.72)	1.29	(0.76-2.17)
Lives with a partner, not with children/grandchildren	171	114	38.2	0.49	(0.34-0.70)	0.46	(0.31-0.69)
Lives with a partner and children/grandchildren	149	90	50.3	0.80	(0.57-1.14)	0.73	(0.49-1.10)
Lives with children/grandchildren, not with a partner	246	94	66.2	1.55	(1.12-2.14)	1.54	(1.08-2.21)
**Attended a GUM clinic in past year**				p<0.001	"	p<0.001	"
Lives with parents or other relatives	860	479	26.8	1.00	"	1.00	"
Lives alone	174	69	23.8	0.85	(0.57-1.28)	0.84	(0.55-1.28)
Lives with non-relatives only	100	100	26.5	0.98	(0.52-1.85)	1.25	(0.65-2.40)
Lives with a partner, not with children/grandchildren	163	109	12.2	0.38	(0.23-0.61)	0.40	(0.23-0.69)
Lives with a partner and children/grandchildren	149	90	8.2	0.25	(0.13-0.45)	0.24	(0.13-0.45)
Lives with children/grandchildren, not with a partner	239	91	21.5	0.75	(0.48-1.16)	0.74	(0.46-1.18)

OR=odds ratio. aAOR=adjusted odds ratio. STI=sexually transmitted infection. GUM=genitourinary medicine. Denominators vary across variables due to item non-response. *Adjusted for age, relationship status, employment/student status and area of residence. †Opposite and/or same-sex partners. §Reported two or more partners of opposite or same sex with no condom use in the past year, or no condom use at first sex with one or more new partners of opposite or same sex in the past year. ‡Diagnosed with chlamydia, gonorrhoea, herpes, genital warts, trichomoniasis, non-specific or non-gonococcal urethritis, or syphilis.

Although women living alone and women living with non-relatives were more likely to report risky sexual behaviours, these groups were not more likely to report chlamydia testing or genitourinary medicine (GUM) clinic attendance in the past year than women living with parents. However, women living with children but not with a partner were more likely to report having tested for chlamydia in the past year (66.2% vs 55.8%; aAOR 1.54 (1.08-2.21)). Women living with partners were less likely than women living with parents to report sexual health service usage (chlamydia testing or GUM clinic attendance).

Among sexually-experienced young men, 65.2% lived with parents or other relatives, 8.3% lived alone, 13.1% lived with non-relatives only, 7.9% lived with a partner but not with children, and 5.5% lived with a partner and children. After adjusting for age, relationship status, employment/student

For peer review only - http://bmjopen.bmj.com/site/about/guidelines.xhtml

status and area of residence, few associations were found between household structure and sexual behaviour and sexual health for men [Table 3]. Compared with men living with parents or other relatives, men living with a partner and no children (11.9% vs 48.9%; aAOR 0.21 (0.10-0.46)) and men living with a partner and children (2.8% vs 48.9%; aAOR 0.04 (0.01-0.18)) were significantly less likely to report having had two or more partners in the past year. Men living with a partner and children were also less likely to have had unsafe sex in the past year (9.6% vs 31.3%; aAOR 0.25 (0.11-0.57)) and to report testing for chlamydia in the past year (20.3% vs 37.1%; aAOR 0.47 (0.24-0.91)).

Table 3: Sexual behaviour and sexual health outcomes in sexually-experienced men aged 16-24 years

	Denominator (unweighted)	Denominator (weighted)	Prevalence (%)	OR	95% CI	aAOR*	95% CI
**SEXUAL BEHAVIOUR**							
**Any sexual intercourse in past 4 weeks**				p<0.001		p=0.49	
Lives with parents or other relatives	922	634	67.3	1.00		1.00	
Lives alone	154	81	77.3	1.66	(1.10-2.50)	1.41	(0.82-2.43)
Lives with non-relatives only	103	127	64.6	0.89	(0.56-1.39)	0.97	(0.56-1.67)
Lives with a partner, not with children/grandchildren	89	79	94.9	9.00	(2.38-34.08)	1.64	(0.42-6.45)
Lives with a partner and children/grandchildren	69	55	96.8	14.58	(3.52-60.42)	2.47	(0.57-10.70)
**Four or more occasions of sexual intercourse in past 4 weeks**				p<0.001		p=0.50	
Lives with parents or other relatives	909	627	36.9	1.00		1.00	
Lives alone	149	78	49.2	1.66	(1.04-2.65)	1.19	(0.60-2.35)
Lives with non-relatives only	101	125	43.3	1.31	(0.85-2.01)	1.43	(0.86-2.36)
Lives with a partner, not with children/grandchildren	86	77	76.5	5.58	(3.18-9.80)	1.45	(0.79-2.66)
Lives with a partner and children/grandchildren	67	53	68.8	3.77	(2.17-6.57)	0.94	(0.52-1.70)
**Two or more partners† in past year**				p<0.001		p<0.001	
Lives with parents or other relatives	932	641	48.9	1.00		1.00	
Lives alone	149	79	50.8	1.08	(0.75-1.56)	1.31	(0.84-2.05)
Lives with non-relatives only	105	129	43.1	0.79	(0.51-1.24)	1.06	(0.67-1.68)
Lives with a partner, not with children/grandchildren	89	79	11.9	0.14	(0.07-0.29)	0.21	(0.10-0.46)
Lives with a partner and children/grandchildren	69	55	2.8	0.03	(0.01-0.13)	0.04	(0.01-0.18)
**Had unsafe sex in past year§**				p=0.006		p=0.009	
Lives with parents or other relatives	931	642	31.3	1.00		1.00	
Lives alone	150	79	33.9	1.12	(0.71-1.77)	1.27	(0.81-1.98)
Lives with non-relatives only	105	129	26.6	0.79	(0.49-1.29)	1.17	(0.69-1.99)
Lives with a partner, not with children/grandchildren	89	79	25.3	0.74	(0.41-1.33)	0.84	(0.45-1.55)
Lives with a partner and children/grandchildren	69	55	9.6	0.23	(0.10-0.52)	0.25	(0.11-0.57)
**SEXUAL HEALTH**							

For peer review only

**Diagnosed with an STI in past year‡**				p=0.92		p=0.98	
Lives with parents or other relatives	935	643	3.1	1.00		1.00	
Lives alone	155	82	2.9	0.94	(0.37-2.37)	1.00	(0.37-2.67)
Lives with non-relatives only	103	126	3.0	0.95	(0.27-3.40)	1.22	(0.28-5.31)
Lives with a partner, not with children/grandchildren	89	79	1.8	0.58	(0.13-2.59)	0.75	(0.15-3.69)
Lives with a partner and children/grandchildren	69	55	0.0	n/a	n/a	n/a	n/a
**Tested for chlamydia in past year**				p=0.03		p=0.10	
Lives with parents or other relatives	941	648	37.1	1.00		1.00	
Lives alone	156	82	35.2	0.92	(0.61-1.39)	0.99	(0.64-1.54)
Lives with non-relatives only	106	130	34.2	0.88	(0.57-1.35)	1.05	(0.65-1.68)
Lives with a partner, not with children/grandchildren	89	79	25.1	0.57	(0.35-0.93)	0.62	(0.36-1.05)
Lives with a partner and children/grandchildren	69	55	20.3	0.43	(0.23-0.81)	0.47	(0.24-0.91)
**Attended a GUM clinic in past year**				p=0.53		p=0.45	
Lives with parents or other relatives	925	637	16.8	1.00		1.00	
Lives alone	155	82	16.7	0.99	(0.57-1.73)	1.16	(0.66-2.05)
Lives with non-relatives only	106	130	19.2	1.18	(0.69-2.03)	1.50	(0.82-2.75)
Lives with a partner, not with children/grandchildren	89	79	16.7	0.99	(0.54-1.81)	1.29	(0.67-2.49)
Lives with a partner and children/grandchildren	66	52	9.4	0.51	(0.23-1.15)	0.64	(0.27-1.50)

OR=odds ratio. aAOR=adjusted odds ratio. STI=sexually transmitted infection. GUM=genitourinary medicine. Denominators vary across variables due to item non-response. Due to low numbers (n=4), statistics on sexual health and sexual behaviour outcomes are not presented for young men living with children/grandchildren but not with a partner. *Adjusted for age, relationship status, employment/student status and area of residence. †Opposite and/or same-sex partners. §Reported two or more partners of opposite or same sex with no condom use in the past year, or no condom use at first sex with one or more new partners of opposite or same sex in the past year. ‡Diagnosed with chlamydia, gonorrhoea, herpes, genital warts, trichomoniasis, non-specific or non-gonococcal urethritis, or syphilis.

DISCUSSION

Using national probability survey data, we identified six distinct types of household structure in the British population, which differed by gender and age-group. Of note, women were more likely than men to live with children but not with a partner, and at older ages, to live alone. We also found that young men were more likely than young women to live with parents, while young women were more likely than young men to live in new family households with a partner and/or children. We found for young women – but not young men – that household structure was associated with reported sexual risk behaviours. Young men and women cohabiting with partners, with or without children, were less likely to report these behaviours than their counterparts who lived with their parents or other relatives. Importantly, these associations were independent of age and relationship

For peer review only

status. Although young women living alone or with non-relatives were more likely to report sexual behaviours linked to STI acquisition,[15] these women were not more likely to report GUM clinic attendance or having tested for chlamydia in the past year. Further adjustment for the number of partners in the past year (not shown) did not remove this association.

Our finding that young women living alone or with non-relatives were more likely than women living with parents to report higher risk sexual behaviours was similar to observations reported in a number of studies of young people in the US which showed that, when compared to young people living at home with parents, those living away from home were more likely to report casual sex,[16, 17] more casual sex partners,[18] inconsistent condom use, and higher-risk sex (including condomless casual sex),[16] and more likely to report having sex while drunk or high.[17] Lyons et al speculated that these differences were due to variations in parental monitoring which allow more opportunities for casual sex,[18] and indeed freedom from parental supervision has been cited as a reason for increased sexual activity in interviews with UK university students,[19] and young seasonal workers employed at holiday centres along the south coast of England.[20] Supporting this qualitative evidence, associations have been found between greater parental knowledge of young people's behaviour and their reporting of fewer sexual partners.[21] Similar associations have been found between parental knowledge of other risk behaviours, including drinking and drug use,[21] and other studies have found associations between living away from parents and increased alcohol use and drug use in young people, especially students.[22, 23] The transition between childhood, marked by dependence on parents or other family members, to adulthood, signified by independence, partnership and parenthood, is recognised as a period of greater risk-taking.[24] A young person's transition route, including the age at which this transition begins, as well as the duration, is dependent on many contextual factors including their gender, educational attainment and socioeconomic status, as well as external factors such as labour market conditions and public policy,[25] all of which are also associated with household structure. Using household structure as an indicator of a young person's life stage thus provides insight into the heterogeneity in

For peer review only

development and lifestyle that exists for this age-group, which should be taken into account when investigating young people's behaviour and health.

Implications for future work

Overall, our study suggests that household structure, as a contextual demographic characteristic, might have an important role in understanding observed differences in sexual behaviour in young women, while playing less of a role for young men. Researchers might therefore consider incorporating household structure information into investigations of the sexual health and behaviour of young people. While this paper focused on young people, it is possible that household structure may also play a role in the sexual behaviour at other life stages. Given the heterogeneity in behaviour that exists across the six household structures we identified, our research also highlights the importance of sampling from a wide range of settings when studying the sexual behaviour and sexual health of young people.

In finding that women who live alone or with non-relatives report higher levels of STI risk behaviour but not greater usage of sexual health services, our study suggests that young women living outside of a family unit, either with parents or a partner, might represent a group with a mismatch between STI risk and sexual healthcare / screening uptake. Clinicians should consider household structure along with other contextual factors when assessing a young person's risk of STIs.

Strengths and limitations

A major strength of this study is that, to our knowledge, it is the first to directly investigate the association between household structure and sexual behaviour and sexual health in young people. In doing so, we used individual-level data from a nationally-representative sample of the British population, in a survey that captured a broad range of sexual health and behaviour outcomes.

While Natsal-3 interviewed over 15,000 women and men aged 16-74 years across Britain and oversampled young people resulting in 2,140 women and 1,729 men aged 16-24 years, it did not oversample ethnic minorities as in Natsal-2.[26] As a reflection of Britain's ethnic composition, the number of participants of non-white British ethnicity was therefore small, and so it was not feasible

For peer review only - http://bmjopen.bmj.com/site/about/guidelines.xhtml

to adjust for ethnicity, which has been shown to be associated with household structure[27] and sexual behaviour,[28] and might therefore represent a confounder that is not accounted for in our analyses. Natsal-3 did not collect information on participants' country of birth, an indicator of migrant status which is also associated with household structure.[6] Due to Natsal's sampling frame, the data are representative of young people living in private households, however young students living at university halls of residence, in institutions or who are homeless are likely to be underrepresented. Although Natsal's study design, including the use of the CASI, is specifically designed to minimise social desirability bias, it remains possible that the presence of parents or other household members at the time of interview may have influenced young participants' willingness to provide accurate answers to sensitive questions.[29, 30] Though young people in Britain are most impacted by STIs,[12] the rates of diagnosed STIs at a population level, and therefore the absolute number of reported diagnoses in our sample, are low. We therefore lacked statistical power to determine differences in STI prevalence by household structure with sufficient precision.

CONCLUSIONS

Household structure information, while routinely collected in surveys, has rarely played a role in epidemiological analyses beyond weighting calculations. Our study suggests that this information may be of more use than previously thought and future research should take account of household structure to further our understanding of the heterogeneity of sexual behaviour. Given the ongoing change in the living situations of young people in Britain, this is particularly pertinent for the sexual health of this key population group.

For peer review only

STATEMENTS

Acknowledgements

The authors thank the study participants, the team of interviewers from NatCen Social Research, operations and computing staff from NatCen Social Research and the study funders. Natsal-3 is a collaboration between University College London (UCL), the London School of Hygiene & Tropical Medicine, NatCen Social Research, Public Health England (formerly the Health Protection Agency), and the University of Manchester.

Contributors

This paper was conceived by TC, NF, SC and CHM. TC wrote the first draft of the article, with further contributions from NF, SC and CHM. TC carried out the statistical analysis, with support from SC, NF and CHM. All authors interpreted data, reviewed successive drafts and approved the final version of the article.

Funding

The study was supported by grants from the Medical Research Council (G0701757 and MR/N013867/1); and the Wellcome Trust (084840), with contributions from the Economic and Social Research Council and Department of Health. Soazig Clifton was funded to undertake independent research supported by the National Institute for Health Research (NIHR Research Methods Programme, Fellowships and Internships, NIHR-RMFI-2014-05-28).

Disclaimer

The sponsors of the study had no role in study design, data collection, data analysis, data interpretation or writing of the report. The corresponding author had full access to all the data in the study and had final responsibility for the decision to submit for publication.

Competing Interests

None

For peer review only

Ethics approval

Natsal-3 was approved by Oxfordshire Research Ethics Committee A (Ref: 09/H0604/27).

Data sharing statement

The Natsal-3 dataset is publicly available from the UK Data Service:

https://discover.ukdataservice.ac.uk/; SN: 7799; persistent identifier: 10.5255/UKDA-SN-77991-1.

Open Access

This is an Open Access article distributed in accordance with the terms of the Creative Commons Attribution (CC BY 4.0) license, which permits others to distribute, remix, adapt and build upon this work, for commercial use, provided the original work is properly cited. See: http://creativecommons.org/licenses/by/4.0/

For peer review only

REFERENCES

1. Copen CE, Chandra A, Febo-Vazquez I. Sexual behavior, sexual attraction, and sexual orientation among adults aged 18-44 in the United States: data from the 2011-2013 National Survey of Family Growth. *Natl Health Stat Report* 2016(88):1-14.
2. Leichliter JS, Chandra A, Liddon N, et al. Prevalence and correlates of heterosexual anal and oral sex in adolescents and adults in the United States. *J Infect Dis* 2007;196(12):1852-9. doi: 10.1086/522867 [published Online First: 15 December 2007]
3. Mercer CH, Jones KG, Johnson AM, et al. How can we objectively categorise partnership type? A novel classification of population survey data to inform epidemiological research and clinical practice. *Sex Transm Infect* 2017;93(2):129-36. doi: 10.1136/sextrans-2016-052646 [published Online First: 17 August 2016]
4. Office for National Statistics. Families and Households. Available from: https://www.ons.gov.uk/peoplepopulationandcommunity/birthsdeathsandmarriages/families/bulletins/familiesandhouseholds/2017 (accessed 15 February 2018).
5. Office for National Statistics. Why are more young people living with their parents? Available from: https://visual.ons.gov.uk/living-with-parents/ (accessed 29 March 2018).
6. Stone J, Berrington A, Falkingham J. The changing determinants of UK young adults' living arrangements. *Demogr Res* 2011;25(20):629-66. doi: 10.4054/DemRes.2011.25.20
7. Bell DNF, Blanchflower DG. Young people and the Great Recession. *Oxford Review of Economic Policy* 2011;27(2):241-67. doi: 10.1093/oxrep/grr011 [published Online First: 01 July 2011]
8. Card D, Lemieux T. Adapting to circumstances: the evolution of work, school, and living arrangements among North American youth. In: Blanchflower DG, Freeman RB, eds. Youth Employment and Joblessness in Advanced Countries. Chicago: University of Chicago Press 2000:171-214.
9. McKee K. Young people, homeownership and future welfare. *Hous Stud* 2012;27(6):853-62. doi: 10.1080/02673037.2012.714463 [published Online First: 13 August 2012]
10. Patton GC, Sawyer SM, Santelli JS, et al. Our future: a Lancet commission on adolescent health and wellbeing. *Lancet* 2016;387(10036):2423-78. doi: 10.1016/S0140-6736(16)00579-1
11. Sonnenberg P, Clifton S, Beddows S, et al. Prevalence, risk factors, and uptake of interventions for sexually transmitted infections in Britain: findings from the National Surveys of Sexual Attitudes and Lifestyles (Natsal). *Lancet* 2013;382(9907):1795-806. doi: 10.1016/S0140-6736(13)61947-9 [published Online First: 26 November 2013]
12. Public Health England. Sexually Transmitted Infections and Chlamydia Screening in England, 2016. Health Protection Report. London: Public Health England, 2017.
13. Mercer CH, Tanton C, Prah P, et al. Changes in sexual attitudes and lifestyles in Britain through the life course and over time: findings from the National Surveys of Sexual Attitudes and Lifestyles (Natsal). *Lancet* 2013;382(9907):1781-94. doi: 10.1016/S0140-6736(13)62035-8 [published Online First: 26 November 2013]
14. Erens B, Phelps A, Clifton S, et al. Methodology of the third British National Survey of Sexual Attitudes and Lifestyles (Natsal-3). *Sex Transm Infect* 2014;90(2):84-9. doi: 10.1136/sextrans-2013-051359 [published Online First: 25 November 2013]
15. Woodhall SC, Soldan K, Sonnenberg P, et al. Is chlamydia screening and testing in Britain reaching young adults at risk of infection? Findings from the third National Survey of Sexual Attitudes and Lifestyles (Natsal-3). *Sex Transm Infect* 2016;92(3):218-27. doi: 10.1136/sextrans-2015-052013 [published Online First: 19 August 2015]
16. Bailey JA, Haggerty KP, White HR, et al. Associations between changing developmental contexts and risky sexual behavior in the two years following high school. *Arch Sex Behav* 2011;40(5):951-60. doi: 10.1007/s10508-010-9633-0 [published Online First: 23 June 2010]
17. Schwartz SJ, Zamboanga BL, Ravert RD, et al. Perceived parental relationships and health-risk behaviors in college-attending emerging adults. *J Marriage Fam* 2009;71(3):727-40. doi: 10.1111/j.1741-3737.2009.00629.x [published Online First: 30 July 2009]

For peer review only

18. Lyons H, Manning W, Giordano P, et al. Predictors of heterosexual casual sex among young adults. *Arch Sex Behav* 2013;42(4):585-93. doi: 10.1007/s10508-012-0051-3 [published Online First: 08 January 2013]
19. Chanakira E, O'Cathain A, Goyder EC, et al. Factors perceived to influence risky sexual behaviours among university students in the United Kingdom: a qualitative telephone interview study. *BMC Public Health* 2014;14:1055. doi: 10.1186/1471-2458-14-1055 [published Online First: 09 October 2014]
20. Hennink M, Cooper P, Diamond I. Seasonal work and sexual behaviour. *J Sex Res* 2000;37(2):175-83. doi: 10.1080/00224490009552035 [published Online First: 11 January 2010]
21. Padilla-Walker LM, Nelson LJ, Madsen SD, et al. The role of perceived parental knowledge on emerging adults' risk behaviors. *J Youth Adolesc* 2008;37(7):847-59. doi: 10.1007/s10964-007-9268-1 [published Online First: 02 February 2008]
22. El Ansari W, Sebena R, Stock C. Socio-demographic correlates of six indicators of alcohol consumption: survey findings of students across seven universities in England, Wales and Northern Ireland. *Arch Public Health* 2013;71:29. doi: 10.1186/2049-3258-71-29 [published Online First: 06 November 2013]
23. El Ansari W, Vallentin-Holbech L, Stock C. Predictors of illicit drug/s use among university students in Northern Ireland, Wales and England. *Glob J Health Sci* 2014;7(4):18-29. doi: 10.5539/gjhs.v7n4p18 [published Online First: 16 December 2014]
24. IOM (Institute of Medicine) and NRC (National Research Council). The Science of Adolescent Risk-Taking: Workshop Report. Washington, DC: Committee on the Science of Adolescence, 2011.
25. Bynner J. British youth transitions in comparative perspective. *J Youth Stud* 2001;4(1):5-23. doi: 10.1080/13676260120028529
26. Fenton KA, Mercer CH, McManus S, et al. Ethnic variations in sexual behaviour in Great Britain and risk of sexually transmitted infections: a probability survey. *Lancet* 2005;365(9466):1246-55. doi: 10.1016/S0140-6736(05)74813-3 [published Online First: 07 April 2005]
27. Finney N. Understanding ethnic differences in the migration of young adults within Britain from a lifecourse perspective. *Transactions of the Institute of British Geographers* 2011;36(3):455-70. doi: 10.1111/j.1475-5661.2011.00426.x [published Online First: 17 March 2011]
28. Wayal S, Hughes G, Sonnenberg P, et al. Ethnic variations in sexual behaviours and sexual health markers: findings from the third British National Survey of Sexual Attitudes and Lifestyles (Natsal-3). *Lancet Public Health* 2017;2(10):e458-e72. doi: 10.1016/S2468-2667(17)30159-7 [published Online First: 24 October 2017]
29. Copas AJ, Wellings K, Erens B, et al. The accuracy of reported sensitive sexual behaviour in Britain: exploring the extent of change 1990–2000. *Sex Transm Infect* 2002;78:26-30. doi: 10.1136/sti.78.1.26
30. Prah P, Copas AJ, Mercer CH, et al. Consistency in reporting sensitive sexual behaviours in Britain: change in reporting bias in the second and third National Surveys of Sexual Attitudes and Lifestyles (Natsal-2 and Natsal-3). *Sex Transm Infect* 2014;90(2):90-3. doi: 10.1136/sextrans-2013-051360 [published Online First: 25 November 2013]

For peer review only

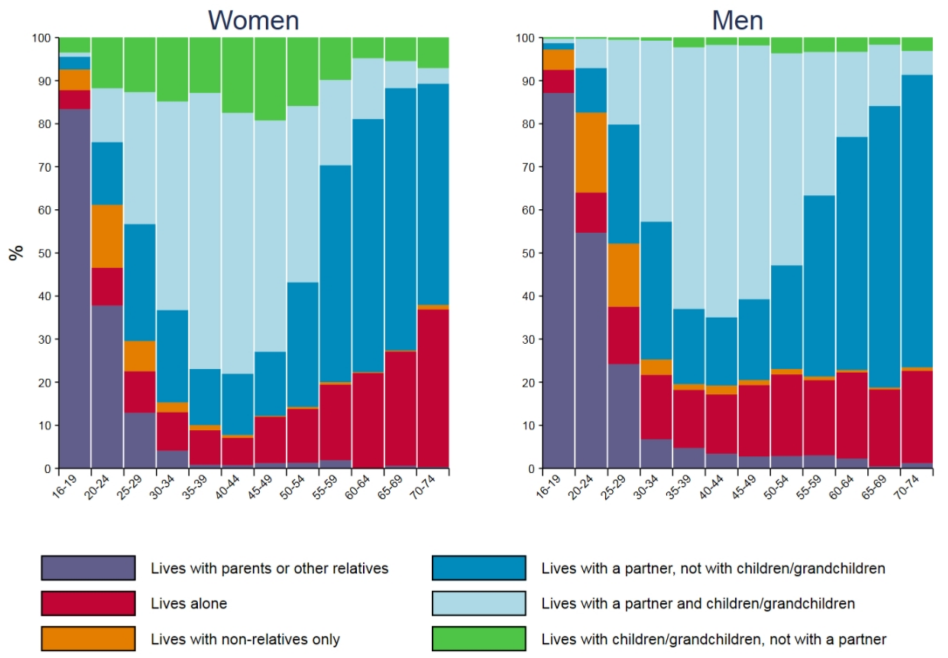

Household structure by gender and age-group among the resident British population aged 16-74 years

512x373mm (72 x 72 DPI)

STROBE (Strengthening The Reporting of OBservational Studies in Epidemiology) Checklist

A checklist of items that should be included in reports of observational studies. You must report the page number in your manuscript where you consider each of the items listed in this checklist. If you have not included this information, either revise your manuscript accordingly before submitting or note N/A.

Note: An Explanation and Elaboration article discusses each checklist item and gives methodological background and published examples of transparent reporting. The STROBE checklist is best used in conjunction with this article (freely available on the Web sites of PLoS Medicine at http://www.plosmedicine.org/, Annals of Internal Medicine at http://www.annals.org/, and Epidemiology at http://www.epidem.com/). Information on the STROBE Initiative is available at www.strobe-statement.org.

Section and Item	Item No.	Recommendation	Reported on Page No.
**Title and Abstract**	1	(*a*) Indicate the study's design with a commonly used term in the title or the abstract	
		(*b*) Provide in the abstract an informative and balanced summary of what was done and what was found	
**Introduction**			
Background/Rationale	2	Explain the scientific background and rationale for the investigation being reported	
Objectives	3	State specific objectives, including any prespecified hypotheses	
**Methods**			
Study Design	4	Present key elements of study design early in the paper	
Setting	5	Describe the setting, locations, and relevant dates, including periods of recruitment, exposure, follow-up, and data collection	
Participants	6	(*a*) *Cohort study*—Give the eligibility criteria, and the sources and methods of selection of participants. Describe methods of follow-up<br>*Case-control study*—Give the eligibility criteria, and the sources and methods of case ascertainment and control selection. Give the rationale for the choice of cases and controls<br>*Cross-sectional study*—Give the eligibility criteria, and the sources and methods of selection of participants	
		(*b*) *Cohort study*—For matched studies, give matching criteria and number of exposed and unexposed<br>*Case-control study*—For matched studies, give matching criteria and the number of controls per case	
Variables	7	Clearly define all outcomes, exposures, predictors, potential confounders, and effect modifiers. Give diagnostic criteria, if applicable	

For peer review only

Section and Item	Item No.	Recommendation	Reported on Page No.
Data Sources/ Measurement	8*	For each variable of interest, give sources of data and details of methods of assessment (measurement). Describe comparability of assessment methods if there is more than one group	
Bias	9	Describe any efforts to address potential sources of bias	
Study Size	10	Explain how the study size was arrived at	
Quantitative Variables	11	Explain how quantitative variables were handled in the analyses. If applicable, describe which groupings were chosen and why	
Statistical Methods	12	(*a*) Describe all statistical methods, including those used to control for confounding	
		(*b*) Describe any methods used to examine subgroups and interactions	
		(*c*) Explain how missing data were addressed	
		(*d*) *Cohort study*—If applicable, explain how loss to follow-up was addressed<br>*Case-control study*—If applicable, explain how matching of cases and controls was addressed<br>*Cross-sectional study*—If applicable, describe analytical methods taking account of sampling strategy	
		(*e*) Describe any sensitivity analyses	
**Results**			
Participants	13*	(a) Report numbers of individuals at each stage of study—eg numbers potentially eligible, examined for eligibility, confirmed eligible, included in the study, completing follow-up, and analysed	
		(b) Give reasons for non-participation at each stage	
		(c) Consider use of a flow diagram	
Descriptive Data	14*	(a) Give characteristics of study participants (eg demographic, clinical, social) and information on exposures and potential confounders	
		(b) Indicate number of participants with missing data for each variable of interest	
		(c) *Cohort study*—Summarise follow-up time (eg, average and total amount)	
Outcome Data	15*	*Cohort study*—Report numbers of outcome events or summary measures over time	
		Case-control study—Report numbers in each exposure category, or summary measures of exposure	
		Cross-sectional study—Report numbers of outcome events or summary measures	

For peer review only

Section and Item	Item No.	Recommendation	Reported on Page No.
Main Results	16	(*a*) Give unadjusted estimates and, if applicable, confounder-adjusted estimates and their precision (eg, 95% confidence interval). Make clear which confounders were adjusted for and why they were included	
		(*b*) Report category boundaries when continuous variables were categorized	
		(*c*) If relevant, consider translating estimates of relative risk into absolute risk for a meaningful time period	
Other Analyses	17	Report other analyses done—eg analyses of subgroups and interactions, and sensitivity analyses	
**Discussion**			
Key Results	18	Summarise key results with reference to study objectives	
Limitations	19	Discuss limitations of the study, taking into account sources of potential bias or imprecision. Discuss both direction and magnitude of any potential bias	
Interpretation	20	Give a cautious overall interpretation of results considering objectives, limitations, multiplicity of analyses, results from similar studies, and other relevant evidence	
Generalisability	21	Discuss the generalisability (external validity) of the study results	
**Other Information**			
Funding	22	Give the source of funding and the role of the funders for the present study and, if applicable, for the original study on which the present article is based	

*Give information separately for cases and controls in case-control studies and, if applicable, for exposed and unexposed groups in cohort and cross-sectional studies.

Once you have completed this checklist, please save a copy and upload it as part of your submission. DO NOT include this checklist as part of the main manuscript document. It must be uploaded as a separate file.

For peer review only

Household structure and its association with sexual risk behaviours and sexual health outcomes: Evidence from a British probability sample survey

Journal:	*BMJ Open*
Manuscript ID	bmjopen-2018-024255.R1
Article Type:	Research
Date Submitted by the Author:	20-Aug-2018
Complete List of Authors:	Curtis, Tyrone; University College London Institute for Global Health, Centre for Population Research in Sexual Health and HIV<br>Field, Nigel; University College London Institute for Global Health, Centre for Molecular Epidemiology and Translational Research<br>Clifton, Soazig; University College London Institute for Global Health, Centre for Population Research in Sexual Health and HIV<br>Mercer, Catherine; University College London Institute for Global Health, Centre for Population Research in Sexual Health and HIV
**Primary Subject Heading**:	Sexual health
Secondary Subject Heading:	Public health, Epidemiology, Research methods
Keywords:	GENITOURINARY MEDICINE, Epidemiology < INFECTIOUS DISEASES, SEXUAL MEDICINE, STATISTICS & RESEARCH METHODS

SCHOLARONE™
Manuscripts

Full title: Household structure and its association with sexual risk behaviours and sexual health outcomes: Evidence from a British probability sample survey

Short title: Household structure and sexual risk behaviour and sexual health

Authors:

Tyrone Curtis*, MSc[a]

Nigel Field, MB PhD[b]

Soazig Clifton, MSc[a]

Catherine H. Mercer, PhD[a]

[a]Centre for Population Research in Sexual Health and HIV, Institute for Global Health, University College London, London, United Kingdom

[b]Centre for Molecular Epidemiology and Translational Research, Institute for Global Health, University College London, London, United Kingdom

***Corresponding Author:** Tyrone Curtis, Centre for Population Research in Sexual Health and HIV, UCL, 3rd Floor, Mortimer Market Centre, off Capper Street, London WC1E 6JB; tyrone.curtis@ucl.ac.uk

Word count: 3373 words

For peer review only

For peer review only - http://bmjopen.bmj.com/site/about/guidelines.xhtml

ABSTRACT

Objectives: Economic and social changes over the last twenty years have led to changes in the living situations of young people in Britain. A person's home-life context might influence their sexual behaviour, with implications for their sexual healthcare needs; we investigated this hypothesis.

Methods: Britain's third National Survey of Sexual Attitudes and Lifestyles (Natsal-3), a probability sample survey undertaken in 2010-2012, interviewed 15,162 men and women aged 16-74 years in Britain (with 3,869 aged 16-24 years). We examined household structure by gender and age-group. We then focused on sexually-experienced young people (aged 16-24 years), and used multivariable models to explore associations between household structure, sexual risk behaviours and sexual health outcomes, independent of confounders including age, relationship status, employment, and area of residence.

Results: Young people were most likely to be living with parents (women 57.1% (95%CI 54.5%-59.6%) and men 68.7% (65.4%-71.8)), or non-relatives (women 10.5% (8.5%-12.9%) and men 12.6% (10.1%-15.6%)). Among the 81.3% of young people who were sexually-experienced, compared with young women living with parents (reference category), those living alone or with non-relatives had a higher likelihood of reporting ≥2 sexual partners (adjusted odds ratio 1.54 (95%CI 1.03-2.31); 1.76 (1.03-3.00), respectively). Women living alone were also more likely to have had unsafe sex (2.04 (1.38-3.02)). Despite these differences in sexually transmitted infection risk, there was no difference in sexual healthcare-seeking behaviour. Young men and women living with partners reported lower levels of sexual risk behaviours.

For peer review only - http://bmjopen.bmj.com/site/about/guidelines.xhtml

Conclusions: Our study suggests household structure may influence the sexual behaviour of young people in Britain. Given changes in their living arrangements, the role of household structure in sexual health research should be further investigated, and also considered as a possible marker for STI risk in clinical consultations.

Total: 285 words

Strengths and limitations of this study

- This is the first study to investigate the association between household structure and sexual behaviour and sexual health among young people; a population group that bears a disproportionate burden of sexual ill-health.
- The study analysed data from a large, national probability sample survey of sexual health and so results can be considered as broadly representative of the British general population.
- The comprehensive nature of the survey enabled us to examine a broad range of sexual risk behaviours and sexual health outcomes.
- The numbers of participants from ethnic minority groups were relatively small, and so it was not possible to adjust for ethnicity in analyses.
- The survey's sampling frame - private households - means that those living in institutions (including some students) or who are homeless are excluded.

For peer review only

INTRODUCTION

Young people in the UK are increasingly spending their early adulthood living with parents.[1] One reason for this may be the increased uptake of higher education, with twice as many students in 2014 living at home for the duration of their studies than in 1996.[2] Those students who do move away from home are increasingly likely to return to the parental home upon completion of their studies, if only for a brief period.[3] Traditionally, a key driver for leaving the parental home has been marriage or, more recently, to cohabit with a partner. However, young people are deferring the age at which they first move in with a partner, and so tend not to form new family units until later in their twenties and early thirties.[2] The living arrangements of young people are also heavily dependent on economic circumstances. The economic uncertainty caused by the financial crisis of 2007-08 and the recession that followed disproportionately affected young people, resulting in high levels of youth unemployment,[4] a factor known to increase the proportion of young people living at home with parents.[5] Restrictions in access to the welfare system and housing market have also made it more likely for young people outside of family households to be living in rented accommodation, often sharing with friends or other non-related adults.[6] Overall, more young people are living at home with their parents, and those who do leave home are more likely to be living for a time outside of a family household.

It is plausible that these changes might affect sexual behaviour, and by implication, sexual health, for example, due to changes in factors identified, through a review guided by the integrative model of behavioural prediction,[7] as acting as constraints on young people's sexual behaviour such as parental monitoring and supervision.[8] Associations have been found between higher-risk sexual behaviour and family structure, that is, the presence of one or both natural parents in the household,[9] as well as levels of parental monitoring[10], however these studies have usually focused on young people below the age of 18. Across the life course, relationship and/or cohabitation status have often been shown to be associated with differences in sexual behaviour[11, 12] and sexual health outcomes.[13] However, to date, there has been no investigation into the

For peer review only - http://bmjopen.bmj.com/site/about/guidelines.xhtml

variations in sexual behaviour and sexual health outcomes according to different types of household structure more broadly, that is, the composition of individuals' households based on their relationships to all other household members, and not just the presence of partners or parents.

Sexual health surveys such as Britain's National Survey of Sexual Attitudes and Lifestyles (Natsal) routinely collect information on participants' household structure. In this study, we used Natsal-3's nationally-representative data to explore the distribution of household structures for the British population aged 16-74 years, and then investigated the independent associations between household structure and the sexual health of young people, specifically young adults aged 16-24 years as the group that experiences the greatest social and sexual transitions[14] and among the highest burden of poor sexual health, including sexually transmitted infections (STIs).[15, 16]

METHODS

Participants and procedures

Full details of the methods of Natsal-3 have been reported elsewhere.[17, 18] Briefly, Natsal-3 was a survey of 15,162 men and women aged 16-74 years resident in Britain, undertaken from 2010 to 2012, and recruited using a multistage, clustered, stratified probability sample design. The response rate was 57.7%, with a co-operation rate (the proportion of interviews completed from eligible addresses for which contact was made) of 65.8%.[18] Included in the sample were 3,869 men and women aged 16-24 years (1,729 men). The Natsal-3 study was approved by the Oxfordshire Research Ethics Committee A (reference: 09/H0604/27). Participants provided oral informed consent for interviews.

Participants were interviewed in their own homes by professional interviewers using a combination of a face-to-face computer-administered personal interview (CAPI) and a computer-assisted self-interview (CASI). Most questions relating to general health and well-being were collected face-to-face. More sensitive questions about participants' sexual behaviour and sexual health, including questions about STI testing and diagnoses, and sexual health clinic attendance, were asked via the

For peer review only - http://bmjopen.bmj.com/site/about/guidelines.xhtml

CASI. Participants were routed into the CASI if they reported sex with an opposite-sex partner and/or reported any sexual experience with someone of the same sex (both since age 13), or if they refused to answer this question. Participants reporting no previous sexual experience did not complete the CASI.

After completing the CASI, participants were asked to provide information on up to 11 additional household members, including their age, sex, and relationship to the participant (partner, son or daughter (including step-, foster or -in-law), parent (including step-, foster or -in-law), brother/sister (including step-, half, foster or -in-law), grandchild, grandparent, other relative or non-relative). Information on other members of participants' households was used to devise six household structure categories (table 1). In the criteria below, "children" and "grandchildren" refer to those of the participant, regardless of age and including step-, foster or -in-law.

Table 1: Household structure categories derived from Natsal-3 questionnaire responses about other members of participants' households

Household structure category	Criteria
Lives with parents or other relatives*	Lives with parents or other relatives (including siblings), but not with a partner or children/grandchildren of their own. Household may include non-relatives.
Lives alone	No other household members.
Lives with non-relatives only	Does not live with parents, other relatives, a partner or children/grandchildren of their own.
Lives with a partner, not with children/grandchildren	Lives with a partner but not with children/grandchildren of their own. Household may include other members.
Lives with a partner and children/grandchildren	Lives with a partner and children/grandchildren of their own. Household may include other members.
Lives with children/grandchildren, not with a partner	Lives with children/grandchildren of their own but does not live with a partner. Household may include other members.

*Participants living with other relatives were grouped with those living with parents recognising that some young people may not live with their parents, for a variety of reasons, and may instead be living with other caretakers such as grandparents or even older siblings, and their relationship to these caretakers may have a

For peer review only - http://bmjopen.bmj.com/site/about/guidelines.xhtml

similar influence on behaviour. Furthermore, only 5.4% (n=164) of all participants (and 3.2% (n=79) of participants aged 16-24 years) in this category did not live with parents.

Patient and Public Involvement

As an unfunded secondary analysis of Natsal-3 data, this particular paper did not entail Patient and Public Involvement. However, public engagement has been a key component of each phase of the (to date) three rounds of the Natsal study, including public consultation exercises to inform new topic areas, cognitive interviews to inform question wording, and interactive public engagement events (including via digital media) to disseminate findings.

Statistical analysis

All analyses were carried out using the complex survey functions of Stata (version 15.1) to incorporate the weighting, clustering and stratification of Natsal-3.[18] We used an alpha of 0.05 in all analyses.

We first calculated the distribution of household structure by gender and age-group for all participants aged 16-74 years (0.6% non-response). To examine how the reporting of sexual risk behaviours and sexual health outcomes by young people varied by household structure category, we used data for 1,717 women and 1,361 men aged 16-24 years who reported having had at least one sexual partner in their lifetime (hereon 'sexually-experienced young people'). We calculated descriptive statistics for each reported outcome, and used multivariate logistic regression to calculate adjusted odds ratios (AORs) to compare participants living with parents or other relatives with those living in other household structure categories.

We examined a number of sexual behaviours linked to STI acquisition[19] and sexual health outcomes, in recognition of the multidimensional nature of sexual health.[20] Sexual intercourse was defined as reporting vaginal, oral or anal sex with a partner of the opposite sex, or oral or anal sex or genital contact with a partner of the same sex. Unsafe sex was defined as reporting two or more partners of opposite or same sex with no condom use in the past year, or no condom use at first sex with one or more new partners of opposite or same sex in the past year. STI diagnosis in the past year was defined as reporting diagnosis with one of the following STIs in the past year:

For peer review only - http://bmjopen.bmj.com/site/about/guidelines.xhtml

trichomoniasis, gonorrhoea, chlamydia, syphilis, non-specific or non-gonococcal urethritis (NSU/NGU), genital warts, or herpes. Other outcomes examined include measures of recency of sex and sexual frequency, reporting two or more partners in the past year, reporting having tested for chlamydia in the past year, and reporting having attended a genitourinary medicine (GUM) clinic in the past year.

In multivariable models, we adjusted for factors in Natsal-3 hypothesised to be associated with both household structure and sexual health. We first adjusted for age and relationship status (categorised as in a steady relationship or not), as the factors most strongly correlated with sexual behaviour, and then further adjusted for area of residence (rural, Greater London, or other urban) and socioeconomic status. To account for variation by socioeconomic status whilst also recognising the difficulty in capturing social class in young people,[21] we used the National Statistics Socio-Economic Classification[22] (categorised here as in employment, in full-time education, or neither). Relationship status was derived from responses to questions about legal marital status as well as the participant's relationship to their most recent sexual partner(s), categorising participants as either in a steady relationship (married, in a civil partnership, in a steady relationship but not married or in a civil partnership) or not. Due to low numbers of participants of non-white ethnicity, it was not feasible to take account of ethnicity in our models.

RESULTS

Variations in household structure by gender and age-group

The majority of people aged 16-74 years in Britain in 2010-12 lived with a partner or a partner and children, with the proportions similar for men (62.6% (61.2%-64.0%)) and women (60.7% (59.6%-61.9%)) (figure 1). However, there were differences by gender for those not living with partners. More women than men were living with children but not with a partner (11.9% (11.1%-12.7%) vs 1.8% (1.4%-2.2%); p<0.001), while more men than women lived with their parents (11.2% (10.6%-11.8%) vs 16.0% (15.2%-17.0%); p<0.001). A greater proportion of older women lived alone; 30.4%

For peer review only - http://bmjopen.bmj.com/site/about/guidelines.xhtml

(27.6%-33.4%) of women aged 65 or older lived alone, compared with 19.3% (16.8%-22.1%) of men of the same age (p<0.001).

Among 16-24-year-olds, fewer women reported living in their parents' home than men; 57.1% (54.5%-59.6%) of women lived with parents or other relatives, compared with 68.7% (65.4%-71.8%) of men of similar age (p<0.001). More women were living in their own family households; 25.6% (23.6%-27.7%) lived with a partner and/or children, compared with 11.1% (9.5%-13.0%) of men (p<0.001). Similar proportions of young men and women reported living in non-family households; 7.6% (6.2%-9.4%) of men and 6.9% (5.9%-8.1%) of women lived alone, and 12.6% (10.1%-15.6%) of men and 10.5% (8.5%-12.9%) of women lived only with non-relatives.

Sample characteristics of sexually-experienced young people (aged 16-24 years) by household structure

Focusing on sexually-experienced young people (80.9% of young women, 81.7% of young men), 50.8% of sexually-experienced young women lived with parents or other relatives, 7.4% lived alone, 10.6% lived with non-relatives only, 11.9% lived with a partner but not with children, 9.4% lived with a partner and children and 9.9% lived with children but not with a partner. Among sexually-experienced young men, 65.2% lived with parents or other relatives, 8.3% lived alone, 13.1% lived with non-relatives only, 7.9% lived with a partner but not with children, and 5.5% lived with a partner and children.

The majority of sexually-experienced young people in our sample were of white ethnicity (88%), and lived in Greater London or other urban areas (table 2). Around 55% were in employment, with a further 37.5% in full-time education. A greater proportion of women than men were in steady relationships (65.6% vs 52.5%, p<0.001). The majority of young people living only with non-relatives were in full-time education (69.5% of women, 76.7% of men), while the majority of those living with partners or partners and children were in employment.

Table 2: Sociodemographic characteristics and household structure of sexually-experienced women and men aged 16-24 years

For peer review only - http://bmjopen.bmj.com/site/about/guidelines.xhtml

WOMEN	Lives with parents or other relatives (%)	Lives alone (%)	Lives with non-relatives only (%)	Lives with a partner, not with children (%)	Lives with a partner and children (%)	Lives with children, not with a partner (%)	Total sample (%)
**Age**							
16-17 years	25.7	6.2	0.9	1.0	0.4	3.1	14.1
18-19 years	29.8	18.2	15.4	11.4	5.1	14.8	21.4
20-24 years	44.5	75.6	83.7	87.7	94.5	82.1	64.5
**Ethnicity**							
White	89.4	82.6	82.5	89.6	85.6	85.4	87.5
Mixed	3.0	5.0	7.2	3.0	4.3	8.4	4.2
Asian/Asian British	2.7	5.9	2.7	2.3	6.5	1.1	3.1
Black/Black British	4.0	5.2	4.4	1.1	2.8	5.2	3.8
Other	0.9	1.3	3.3	4.0	0.8	0.0	1.4
**Employment/student status**							
In employment	47.7	54.9	27.0	76.7	82.6	68.8	54.9
In full-time education	46.2	31.5	69.5	17.4	2.4	10.4	36.5
Not in employment or education	6.1	13.7	3.5	5.9	15.0	20.8	8.7
**Area of residence**							
Rural	20.7	11.3	3.7	24.3	14.0	15.9	17.5
Greater London	11.8	20.3	9.7	7.8	4.9	15.5	11.4
Other urban	67.5	68.4	86.6	68.0	81.1	68.6	71.0
**Relationship status**							
Not in a steady relationship	43.6	38.2	49.3	0.0	0.0	42.9	34.3
In a steady relationship	56.4	61.8	50.7	100.0	100.0	57.1	65.6
**Denominators**							
Unweighted total (N)	872	178	101	171	149	246	1717
Weighted total (N)	486	71	101	114	90	94	956
Total (%) (weighted)	50.8	7.4	10.6	11.9	9.4	9.9	

MEN	Lives with parents or other relatives (%)	Lives alone (%)	Lives with non-relatives only (%)	Lives with a partner, not with children (%)	Lives with a partner and children (%)	Lives with children, not with a partner (%)	Total sample (%)
**Age**							
16-17 years	21.7	2.8	0.0	0.0	2.2	-	14.5
18-19 years	26.3	25.0	17.0	10.2	8.7	-	22.7
20-24 years	52.0	72.2	83.0	89.8	89.1	-	62.8
**Ethnicity**							
White	86.7	89.7	83.4	98.0	96.0	-	87.9
Mixed	3.8	0.9	3.7	0.0	0.0	-	3.0
Asian/Asian British	5.2	3.4	3.7	1.0	3.1	-	4.4
Black/Black British	3.2	2.1	7.3	1.0	0.0	-	3.3
Other	1.1	3.8	1.9	0.0	1.0	-	1.3
**Employment/student status**							
In employment	54.9	58.5	23.3	85.7	94.3	-	55.7
In full-time education	37.5	33.4	76.7	12.5	4.2	-	38.5
Not in employment or education	7.6	8.1	0.0	1.8	1.5	-	5.8
**Area of residence**							
Rural	22.2	9.3	4.3	13.5	23.0	-	18.2
Greater London	13.6	7.8	15.6	5.2	3.0	-	12.1
Other urban	64.1	82.9	80.1	81.3	74.1	-	69.7
**Relationship status**							
Not in a steady relationship	57.2	43.2	51.2	0.0	0.0	-	47.5
In a steady relationship	42.9	56.8	48.8	100.0	100.0	-	52.5
**Denominators**							
Unweighted total (N)	941	156	106	89	69	4	1365
Weighted total (N)	648	82	130	79	55	-	994
Total (%) (weighted)	65.2	8.3	13.1	7.9	5.5	-	

Figures presented are column percentages. *Due to low numbers (n=4), statistics are not presented for young men living with children but not with a partner.

For peer review only

Variations in sexual risk behaviours and sexual health outcomes reported by sexually-experienced young people (aged 16-24 years) by household structure

Compared with young women living with parents or other relatives, more of those living alone, living with partners but not with children, or living with partners and children reported having had sex in the past 4 weeks (weighted prevalence 74.0% vs 83.7%, 97.7% and 90.2% respectively) (table 3). After adjusting for age, relationship status, area of residence and employment/student status, women living alone and women living with partners but not with children remained more likely to report having had recent sex than their counterparts living with parents or other relatives (AOR 1.84 (95% CI 1.11-3.04) and 3.32 (1.13-9.74) respectively). Similarly, women living alone (45.5% vs 40.7%; AOR 1.54 (1.03-2.31)) or with non-relatives (47.5% vs 40.7%; AOR 1.76 (1.03-3.00)) were more likely to report two or more partners in the past year. Women living alone were also more likely to have had unsafe sex in the past year (44.2% vs 32.7%; AOR 2.04 (1.38-3.02)).

For peer re

Table 3: Sexual risk behaviours and sexual health outcomes in sexually-experienced women aged 16-24 years

	Denominator (unweighted)	Denominator (weighted)	Prevalence (%)	OR	95% CI	Partially adjusted OR*	95% CI	Fully adjusted OR**	95% CI
**Any sexual intercourse in past 4 weeks**				p<0.001		p=0.02		p=0.02	
Lives with parents or other relatives	849	472	74.0	1.00		1.00		1.00	
Lives alone	176	70	83.7	1.80	(1.18-2.75)	1.87	(1.15 - 3.06)	1.84	(1.11-3.04)
Lives with non-relatives only	96	95	71.8	0.89	(0.54-1.47)	0.98	(0.51 - 1.88)	0.99	(0.51-1.90)
Lives with a partner, not with children	169	112	97.7	14.81	(5.37-40.86)	3.34	(1.14 - 9.78)	3.32	(1.13-9.74)
Lives with a partner and children	147	89	90.2	3.24	(1.76-5.99)	0.74	(0.37 - 1.45)	0.69	(0.35-1.38)
Lives with children, not with a partner	241	93	74.1	1.00	(0.68-1.47)	1.02	(0.64 - 1.61)	0.96	(0.60-1.53)
**Four or more occasions of sexual intercourse in past 4 weeks**				p<0.001		p=0.41		p=0.32	
Lives with parents or other relatives	810	453	45.3	1.00		1.00		1.00	
Lives alone	158	63	53.2	1.37	(0.94-2.00)	1.35	(0.87 - 2.11)	1.41	(0.89-2.23)
Lives with non-relatives only	90	90	44.5	0.97	(0.62-1.52)	1.14	(0.71 - 1.84)	1.21	(0.75-1.96)
Lives with a partner, not with children	151	101	67.2	2.47	(1.70-3.61)	1.08	(0.71 - 1.66)	1.03	(0.68-1.58)
Lives with a partner and children	137	83	60.9	1.88	(1.28-2.78)	0.84	(0.53 - 1.31)	0.80	(0.50-1.26)
Lives with children, not with a partner	224	86	40.5	0.82	(0.59-1.15)	0.80	(0.52 - 1.25)	0.81	(0.52-1.27)
**Two or more partners† in past year**				p<0.001		p<0.001		p<0.001	
Lives with parents or other relatives	866	483	40.7	1.00		1.00		1.00	
Lives alone	176	70	45.5	1.22	(0.84-1.77)	1.47	(1.00 - 2.17)	1.54	(1.03-2.31)
Lives with non-relatives only	99	99	47.5	1.32	(0.81-2.17)	1.50	(0.91 - 2.50)	1.76	(1.03-3.00)
Lives with a partner, not with children	169	112	9.9	0.16	(0.09-0.27)	0.29	(0.16 - 0.52)	0.28	(0.16-0.49)
Lives with a partner and children	148	90	6.5	0.10	(0.05-0.20)	0.19	(0.09 - 0.38)	0.17	(0.09-0.35)
Lives with children, not with a partner	245	94	37.3	0.87	(0.63-1.20)	1.03	(0.72 - 1.48)	1.00	(0.69-1.43)
**Had unsafe sex in past year§**				p<0.001		p<0.001		p<0.001	
Lives with parents or other relatives	863	482	32.7	1.00		1.00		1.00	
Lives alone	176	70	44.2	1.63	(1.12-2.38)	2.06	(1.39 - 3.03)	2.04	(1.38-3.02)
Lives with non-relatives only	98	96	26.6	0.75	(0.44-1.28)	0.93	(0.54 - 1.60)	1.07	(0.60-1.92)
Lives with a partner, not with children	169	112	12.1	0.28	(0.17-0.46)	0.49	(0.29 - 0.83)	0.48	(0.29-0.81)
Lives with a partner and children	149	90	14.9	0.36	(0.22-0.58)	0.65	(0.38 - 1.10)	0.62	(0.36-1.06)
Lives with children, not with a partner	246	94	35.8	1.15	(0.83-1.59)	1.49	(1.04 - 2.12)	1.41	(0.98-2.04)
**Diagnosed with an STI in past year‡**				p=0.19		p=0.19		p=0.13	

For peer review only - http://bmjopen.bmj.com/site/about/guidelines.xhtml

Lives with parents or other relatives	869	484	5.1	1.00		1.00		1.00	
Lives alone	178	71	5.9	1.17	(0.53-2.57)	1.03	(0.43 - 2.49)	1.05	(0.41-2.68)
Lives with non-relatives only	101	101	4.8	0.93	(0.21-4.04)	0.80	(0.17 - 3.84)	0.94	(0.21-4.17)
Lives with a partner, not with children	170	113	3.3	0.64	(0.26-1.53)	0.51	(0.17 - 1.54)	0.51	(0.17-1.49)
Lives with a partner and children	149	90	0.8	0.16	(0.04-0.69)	0.12	(0.02 - 0.66)	0.12	(0.02-0.61)
Lives with children, not with a partner	244	94	4.3	0.83	(0.41-1.65)	0.70	(0.30 - 1.64)	0.67	(0.27-1.67)
**Tested for chlamydia in past year**				p<0.001		p<0.001		p<0.001	
Lives with parents or other relatives	870	485	55.8	1.00		1.00		1.00	
Lives alone	178	71	55.1	0.97	(0.67-1.41)	1.00	(0.67 - 1.47)	0.96	(0.65-1.43)
Lives with non-relatives only	101	101	57.1	1.05	(0.64-1.72)	1.12	(0.67 - 1.86)	1.29	(0.76-2.17)
Lives with a partner, not with children	171	114	38.2	0.49	(0.34-0.70)	0.46	(0.31 - 0.70)	0.46	(0.31-0.69)
Lives with a partner and children	149	90	50.3	0.80	(0.57-1.14)	0.77	(0.52 - 1.14)	0.73	(0.49-1.10)
Lives with children, not with a partner	246	94	66.2	1.55	(1.12-2.14)	1.64	(1.16 - 2.31)	1.54	(1.08-2.21)
**Attended a GUM clinic in past year**				p<0.001		p<0.001		p<0.001	
Lives with parents or other relatives	860	479	26.8	1.00		1.00		1.00	
Lives alone	174	69	23.8	0.85	(0.57-1.28)	0.89	(0.59 - 1.36)	0.84	(0.55-1.28)
Lives with non-relatives only	100	100	26.5	0.98	(0.52-1.85)	1.05	(0.55 - 2.00)	1.25	(0.65-2.40)
Lives with a partner, not with children	163	109	12.2	0.38	(0.23-0.61)	0.39	(0.23 - 0.68)	0.40	(0.23-0.69)
Lives with a partner and children	149	90	8.2	0.25	(0.13-0.45)	0.26	(0.14 - 0.49)	0.24	(0.13-0.45)
Lives with children, not with a partner	239	91	21.5	0.75	(0.48-1.16)	0.80	(0.51 - 1.26)	0.74	(0.46-1.18)

OR=odds ratio. STI=sexually transmitted infection. GUM=genitourinary medicine. Denominators vary across outcomes due to item non-response. *Adjusted for age and relationship status. **Adjusted for age, relationship status, employment/student status and area of residence. †Opposite and/or same-sex partners. §Reported two or more partners of opposite or same sex with no condom use in the past year, or no condom use at first sex with one or more new partners of opposite or same sex in the past year. ‡Diagnosed with chlamydia, gonorrhoea, herpes, genital warts, trichomoniasis, non-specific or non-gonococcal urethritis, or syphilis.

Although women living alone and women living with non-relatives were more likely to report sexual risk behaviours, these groups were not more likely to report chlamydia testing or GUM clinic attendance in the past year than women living with parents. However, women living with children but not with a partner were more likely to report having tested for chlamydia in the past year (66.2% vs 55.8%; AOR 1.54 (1.08-2.21)). Women living with partners were less likely than women living with parents to report sexual health service usage (chlamydia testing or GUM clinic attendance).

After adjusting for age, relationship status, employment/student status and area of residence, few associations were found between household structure and sexual risk behaviours and sexual health outcomes for men (table 4). Compared with men living with parents or other relatives, men living with a partner and no children (11.9% vs 48.9%; AOR 0.21 (0.10-0.46)) and men living with a partner and children (2.8% vs 48.9%; AOR 0.04 (0.01-0.18)) were significantly less likely to report having had two or more partners in the past year. Men living with a partner and children were also less likely to

For peer review only - http://bmjopen.bmj.com/site/about/guidelines.xhtml

have had unsafe sex in the past year (9.6% vs 31.3%; AOR 0.25 (0.11-0.57)) and to report testing for chlamydia in the past year (20.3% vs 37.1%; AOR 0.47 (0.24-0.91)).

Table 4: Sexual risk behaviours and sexual health outcomes in sexually-experienced men aged 16-24 years

	Denominator (unweighted)	Denominator (weighted)	Prevalence (%)	OR	95% CI	Partially adjusted OR*	95% CI	Fully adjusted OR**	95% CI
**Any sexual intercourse in past 4 weeks**				p<0.001		p=0.24		p=0.49	
Lives with parents or other relatives	922	634	67.3	1.00		1.00		1.00	
Lives alone	154	81	77.3	1.66	(1.10-2.50)	1.34	(0.78 - 2.31)	1.41	(0.82-2.43)
Lives with non-relatives only	103	127	64.6	0.89	(0.56-1.39)	0.74	(0.44 - 1.24)	0.97	(0.56-1.67)
Lives with a partner, not with children	89	79	94.9	9.00	(2.38-34.08)	1.65	(0.42 - 6.47)	1.64	(0.42-6.45)
Lives with a partner and children	69	55	96.8	14.58	(3.52-60.42)	2.68	(0.62 - 11.59)	2.47	(0.57-10.70)
**Four or more occasions of sexual intercourse in past 4 weeks**				p<0.001		p=0.77		p=0.50	
Lives with parents or other relatives	909	627	36.9	1.00		1.00		1.00	
Lives alone	149	78	49.2	1.66	(1.04-2.65)	1.15	(0.55 - 2.40)	1.19	(0.60-2.35)
Lives with non-relatives only	101	125	43.3	1.31	(0.85-2.01)	1.13	(0.70 - 1.84)	1.43	(0.86-2.36)
Lives with a partner, not with children	86	77	76.5	5.58	(3.18-9.80)	1.48	(0.81 - 2.72)	1.45	(0.79-2.66)
Lives with a partner and children	67	53	68.8	3.77	(2.17-6.57)	1.00	(0.55 - 1.82)	0.94	(0.52-1.70)
**Two or more partners† in past year**				p<0.001		p<0.001		p<0.001	
Lives with parents or other relatives	932	641	48.9	1.00		1.00		1.00	
Lives alone	149	79	50.8	1.08	(0.75-1.56)	1.20	(0.79 - 1.83)	1.31	(0.84-2.05)
Lives with non-relatives only	105	129	43.1	0.79	(0.51-1.24)	0.81	(0.52 - 1.25)	1.06	(0.67-1.68)
Lives with a partner, not with children	89	79	11.9	0.14	(0.07-0.29)	0.21	(0.10 - 0.47)	0.21	(0.10-0.46)
Lives with a partner and children	69	55	2.8	0.03	(0.01-0.13)	0.05	(0.01 - 0.19)	0.04	(0.01-0.18)
**Had unsafe sex in past year§**				p=0.006		p=0.02		p=0.009	
Lives with parents or other relatives	931	642	31.3	1.00		1.00		1.00	
Lives alone	150	79	33.9	1.12	(0.71-1.77)	1.14	(0.72 - 1.81)	1.27	(0.81-1.98)
Lives with non-relatives only	105	129	26.6	0.79	(0.49-1.29)	0.80	(0.49 - 1.30)	1.17	(0.69-1.99)
Lives with a partner, not with children	89	79	25.3	0.74	(0.41-1.33)	0.81	(0.44 - 1.49)	0.84	(0.45-1.55)
Lives with a partner and children	69	55	9.6	0.23	(0.10-0.52)	0.25	(0.11 - 0.59)	0.25	(0.11-0.57)
**Diagnosed with an STI in past year‡**				p=0.92		p=0.99		p=0.98	
Lives with parents or other relatives	935	643	3.1	1.00		1.00		1.00	
Lives alone	155	82	2.9	0.94	(0.37-2.37)	0.96	(0.37 - 2.51)	1.00	(0.37-2.67)
Lives with non-relatives only	103	126	3.0	0.95	(0.27-3.40)	0.94	(0.25 - 3.44)	1.22	(0.28-5.31)
Lives with a partner, not with children	89	79	1.8	0.58	(0.13-2.59)	0.77	(0.15 - 3.87)	0.75	(0.15-3.69)
Lives with a partner and children	69	55	0.0	n/a	n/a	n/a	n/a	n/a	n/a
**Tested for chlamydia in past year**				p=0.03		p=0.13		p=0.10	
Lives with parents or other relatives	941	648	37.1	1.00		1.00		1.00	
Lives alone	156	82	35.2	0.92	(0.61-1.39)	0.99	(0.65 - 1.52)	0.99	(0.64-1.54)
Lives with non-relatives only	106	130	34.2	0.88	(0.57-1.35)	0.95	(0.62 - 1.48)	1.05	(0.65-1.68)
Lives with a partner, not with children	89	79	25.1	0.57	(0.35-0.93)	0.62	(0.37 - 1.07)	0.62	(0.36-1.05)
Lives with a partner and children	69	55	20.3	0.43	(0.23-0.81)	0.47	(0.24 - 0.92)	0.47	(0.24-0.91)
**Attended a GUM clinic in past year**				p=0.53		p=0.64		p=0.45	
Lives with parents or other relatives	925	637	16.8	1.00		1.00		1.00	
Lives alone	155	82	16.7	0.99	(0.57-1.73)	1.10	(0.62 - 1.95)	1.16	(0.66-2.05)
Lives with non-relatives only	106	130	19.2	1.18	(0.69-2.03)	1.28	(0.73 - 2.24)	1.50	(0.82-2.75)
Lives with a partner, not with children	89	79	16.7	0.99	(0.54-1.81)	1.29	(0.67 - 2.49)	1.29	(0.67-2.49)
Lives with a partner and children	66	52	9.4	0.51	(0.23-1.15)	0.67	(0.28 - 1.57)	0.64	(0.27-1.50)

OR=odds ratio. STI=sexually transmitted infection. GUM=genitourinary medicine. Denominators vary across outcomes due to item non-response. Due to low numbers (n=4), statistics on sexual health outcomes and sexual risk behaviours are not presented for young men living with children but not with a partner. *Adjusted for age and relationship status. **Adjusted for age, relationship status, employment/student status and area of residence. †Opposite and/or same-sex partners. §Reported two or more partners of opposite or same sex with no condom use in the past year, or no condom use at first sex with one or more new partners of opposite or same sex in the past year. ‡Diagnosed with chlamydia, gonorrhoea, herpes, genital warts, trichomoniasis, non-specific or non-gonococcal urethritis, or syphilis.

For peer review only - http://bmjopen.bmj.com/site/about/guidelines.xhtml

DISCUSSION

Using national probability survey data, we identified six distinct types of household structure in the British population, which differed by gender and age-group. Of note, women were more likely than men to live with children but not with a partner, and at older ages, to live alone. We also found that young men were more likely than young women to live with parents, while young women were more likely than young men to live in new family households with a partner and/or children. We found for both young men and women that household structure was associated with reporting sexual risk behaviours. Young men and women cohabiting with partners, with or without children, were less likely to report these behaviours than their counterparts who lived with their parents or other relatives, while young women living alone or with non-relatives were more likely to report these behaviours. Importantly, these associations were independent of age and relationship status. Although young women living outside of family households were more likely to report sexual behaviours linked to STI acquisition, these women were not more likely to report GUM clinic attendance or having tested for chlamydia in the past year. Further adjustment for the number of partners in the past year (not shown) did not remove this association.

Our finding that young women living alone or with non-relatives were more likely than women living with parents to report sexual risk behaviours was similar to those of a number of studies of young people in the US which showed that, when compared to young people living at home with parents, those living away from home were more likely to report casual sex,[23, 24] more casual sex partners,[25] inconsistent condom use, and higher-risk sex (including condomless casual sex),[23] and more likely to report having sex while drunk or high.[24] Lyons et al speculated that these differences were due to variations in parental monitoring which allow more opportunities for casual sex,[25] and indeed freedom from parental supervision was cited as a reason for increased sexual activity in interviews with UK university students.[26] Our finding of associations between living away from parents and higher reporting of sexual risk behaviours in young women but not in young men is supported by evidence from a large study of Scottish teenagers which found that a low level

For peer review only - http://bmjopen.bmj.com/site/about/guidelines.xhtml

For peer review only

of parental monitoring was associated with early sexual activity for both men and women, but additionally for women with higher numbers of sexual partners, and inconsistent condom and contraceptive use.[10] Similar associations have been found between parental knowledge of other risk behaviours, including drinking and drug use,[27] and other studies have found associations between living away from parents and increased alcohol use and drug use in young people, especially students.[28, 29] Additionally, we found associations with sexual risk behaviours for women living alone that were not found for women living with non-relatives, perhaps indicating that for young women, living with others – regardless of who - moderates sexual risk behaviour.

The transition between childhood, marked by dependence on parents or other family members, to adulthood, signified by independence, partnership and parenthood, is recognised as a period of greater risk-taking.[30] A young person's transition route, including the age at which this transition begins, as well as the duration, is dependent on many contextual factors including their gender, educational attainment and socioeconomic status, as well as external factors such as labour market conditions and public policy,[31] all of which are also associated with household structure. Using household structure as an indicator of a young person's life stage thus provides insight into the heterogeneity in development and lifestyle that exists for this age-group, which should be taken into account when investigating young people's behaviour and health.

Implications for future work

Overall, our study suggests that household structure, as a contextual demographic characteristic, might have an important role in understanding observed differences in sexual risk behaviours in young women, while playing less of a role for young men. Researchers might therefore consider incorporating household structure information into investigations of the sexual health and behaviour of young people. While this paper focused on young people, it is plausible that household structure may also play a role in sexual behaviour at other life stages. Given the heterogeneity in behaviour that exists across the six household structures we identified, our research also highlights

For peer review only - http://bmjopen.bmj.com/site/about/guidelines.xhtml

the importance of sampling from a wide range of settings when studying the sexual behaviour and sexual health of young people.

In finding that women who live alone or with non-relatives report higher levels of sexual risk behaviours but not greater usage of sexual health services, our study suggests that young women living outside of a family unit, neither with parents nor a partner, might represent a group with a mismatch between STI risk and sexual healthcare / screening uptake. Clinicians should therefore perhaps consider household structure along with other contextual factors when assessing a young person's risk of STIs.

Strengths and limitations

A major strength of this study is that, to our knowledge, it is the first to directly investigate the association between household structure and sexual risk behaviour and sexual health in young people. In doing so, we used individual-level data from a nationally-representative sample of the British population, in a survey that captured a broad range of sexual health and behaviour outcomes.

While Natsal-3 interviewed over 15,000 women and men aged 16-74 years across Britain and oversampled young people resulting in 2,140 women and 1,729 men aged 16-24 years, it did not oversample ethnic minorities as in Natsal-2.[32] As a reflection of Britain's ethnic composition, the number of participants of non-white British ethnicity was therefore small, and so it was not feasible to adjust for ethnicity, which has been shown to be associated with household structure[33] and sexual behaviour,[34] and might therefore represent a confounder that we were unable to account for in our analyses. Natsal-3 did not collect information on participants' country of birth, an indicator of migrant status which is also associated with household structure.[3] Due to Natsal's sampling frame, the data are representative of young people living in private households, however young students living at university halls of residence, in institutions or who are homeless are likely to be underrepresented. Although Natsal's study design, including the use of the CASI, is specifically designed to minimise social desirability bias, it remains possible that the presence of parents or other household members at the time of interview may have influenced young participants'

For peer review only - http://bmjopen.bmj.com/site/about/guidelines.xhtml

willingness to provide accurate answers to sensitive questions.[35, 36] Though young people in Britain are most impacted by STIs,[16] the rates of diagnosed STIs at a population level, and therefore the absolute number of reported diagnoses in our sample, are relatively low. We therefore lacked statistical power to determine differences in reporting prior STI diagnoses by household structure with sufficient precision.

CONCLUSIONS

Economic and social changes over the last twenty years have led to changes in the living situations of young people in Britain. Our study suggests that household structure may play a role in influencing their sexual risk behaviour. Future research should therefore take account of household structure to further our understanding of the heterogeneity of sexual behaviour, and by implication, sexual health of this key population group.

For peer review only

For peer review only - http://bmjopen.bmj.com/site/about/guidelines.xhtml

STATEMENTS

Acknowledgements

The authors thank the study participants, the team of interviewers from NatCen Social Research, operations and computing staff from NatCen Social Research and the study funders. Natsal-3 is a collaboration between University College London (UCL), the London School of Hygiene & Tropical Medicine, NatCen Social Research, Public Health England (formerly the Health Protection Agency), and the University of Manchester.

Contributors

This paper was conceived by TC, NF, SC and CHM. TC wrote the first draft of the article, with further contributions from NF, SC and CHM. TC carried out the statistical analysis, with support from SC, NF and CHM. All authors interpreted data, reviewed successive drafts and approved the final version of the article.

Funding

The study was supported by grants from the Medical Research Council (G0701757 and MR/N013867/1); and the Wellcome Trust (084840), with contributions from the Economic and Social Research Council and Department of Health. Soazig Clifton was funded to undertake independent research supported by the National Institute for Health Research (NIHR Research Methods Programme, Fellowships and Internships, NIHR-RMFI-2014-05-28).

Disclaimer

The sponsors of the study had no role in study design, data collection, data analysis, data interpretation or writing of the report. The corresponding author had full access to all the data in the study and had final responsibility for the decision to submit for publication.

Competing Interests

None

For peer review only

For peer review only - http://bmjopen.bmj.com/site/about/guidelines.xhtml

Ethics approval

Natsal-3 was approved by Oxfordshire Research Ethics Committee A (Ref: 09/H0604/27).

Data sharing statement

The Natsal-3 dataset is publicly available from the UK Data Service:

https://discover.ukdataservice.ac.uk/; SN: 7799; persistent identifier: 10.5255/UKDA-SN-77991-1.

Open Access

This is an Open Access article distributed in accordance with the terms of the Creative Commons Attribution (CC BY 4.0) license, which permits others to distribute, remix, adapt and build upon this work, for commercial use, provided the original work is properly cited. See: http://creativecommons.org/licenses/by/4.0/

For peer review only

REFERENCES

1. Office for National Statistics. Families and Households. Available from: https://www.ons.gov.uk/peoplepopulationandcommunity/birthsdeathsandmarriages/families/bulletins/familiesandhouseholds/2017 (accessed 15 February 2018).
2. Office for National Statistics. Why are more young people living with their parents? Available from: https://visual.ons.gov.uk/living-with-parents/ (accessed 29 March 2018).
3. Stone J, Berrington A, Falkingham J. The changing determinants of UK young adults' living arrangements. *Demogr Res* 2011;25(20):629-66. doi: 10.4054/DemRes.2011.25.20
4. Bell DNF, Blanchflower DG. Young people and the Great Recession. *Oxford Review of Economic Policy* 2011;27(2):241-67. doi: 10.1093/oxrep/grr011 [published Online First: 01 July 2011]
5. Card D, Lemieux T. Adapting to circumstances: the evolution of work, school, and living arrangements among North American youth. In: Blanchflower DG, Freeman RB, eds. Youth Employment and Joblessness in Advanced Countries. Chicago: University of Chicago Press 2000:171-214.
6. McKee K. Young people, homeownership and future welfare. *Hous Stud* 2012;27(6):853-62. doi: 10.1080/02673037.2012.714463 [published Online First: 13 August 2012]
7. Fishbein, M., Ajzen, I. (2010). Predicting and Changing Behavior. New York: Psychology Press.
8. Buhi ER, Goodson P. Predictors of adolescent sexual behavior and intention: a theory-guided systematic review. *J Adolesc Health* 2007;40(1):4-21. doi: 10.1016/j.jadohealth.2006.09.027 [published Online First: 23 December 2006]
9. Bonell C, Allen E, Strange V, et al. Influence of family type and parenting behaviours on teenage sexual behaviour and conceptions. *J Epidemiol Community Health* 2006;60(6):502-6. doi: 10.1136/jech.2005.042838 [published Online First: 16 May 2006]
10. Wight D, Williamson L, Henderson M. Parental influences on young people's sexual behaviour: a longitudinal analysis. *J Adolesc* 2006;29(4):473-94. doi: 10.1016/j.adolescence.2005.08.007 [published Online First: 11 October 2005]
11. Copen CE, Chandra A, Febo-Vazquez I. Sexual behavior, sexual attraction, and sexual orientation among adults aged 18-44 in the United States: data from the 2011-2013 National Survey of Family Growth. *Natl Health Stat Report* 2016(88):1-14.
12. Leichliter JS, Chandra A, Liddon N, et al. Prevalence and correlates of heterosexual anal and oral sex in adolescents and adults in the United States. *J Infect Dis* 2007;196(12):1852-9. doi: 10.1086/522867 [published Online First: 15 December 2007]
13. Mercer CH, Jones KG, Johnson AM, et al. How can we objectively categorise partnership type? A novel classification of population survey data to inform epidemiological research and clinical practice. *Sex Transm Infect* 2017;93(2):129-36. doi: 10.1136/sextrans-2016-052646 [published Online First: 17 August 2016]
14. Patton GC, Sawyer SM, Santelli JS, et al. Our future: a Lancet commission on adolescent health and wellbeing. *Lancet* 2016;387(10036):2423-78. doi: 10.1016/S0140-6736(16)00579-1
15. Sonnenberg P, Clifton S, Beddows S, et al. Prevalence, risk factors, and uptake of interventions for sexually transmitted infections in Britain: findings from the National Surveys of Sexual Attitudes and Lifestyles (Natsal). *Lancet* 2013;382(9907):1795-806. doi: 10.1016/S0140-6736(13)61947-9 [published Online First: 26 November 2013]
16. Public Health England. Sexually Transmitted Infections and Chlamydia Screening in England, 2016. Health Protection Report. London: Public Health England, 2017.
17. Mercer CH, Tanton C, Prah P, et al. Changes in sexual attitudes and lifestyles in Britain through the life course and over time: findings from the National Surveys of Sexual Attitudes and Lifestyles (Natsal). *Lancet* 2013;382(9907):1781-94. doi: 10.1016/S0140-6736(13)62035-8 [published Online First: 26 November 2013]
18. Erens B, Phelps A, Clifton S, et al. Methodology of the third British National Survey of Sexual Attitudes and Lifestyles (Natsal-3). *Sex Transm Infect* 2014;90(2):84-9. doi: 10.1136/sextrans-2013-051359 [published Online First: 25 November 2013]

For peer review only - http://bmjopen.bmj.com/site/about/guidelines.xhtml

19. Woodhall SC, Soldan K, Sonnenberg P, et al. Is chlamydia screening and testing in Britain reaching young adults at risk of infection? Findings from the third National Survey of Sexual Attitudes and Lifestyles (Natsal-3). *Sex Transm Infect* 2016;92(3):218-27. doi: 10.1136/sextrans-2015-052013 [published Online First: 19 August 2015]
20. World Health Organisation. Defining sexual health: report of a technical consultation on sexual health, 28–31 January 2002, Geneva. Sexual health document series. Geneva: World Health Organisation, 2006.
21. Sheringham J, Mann S, Simms I, et al. It matters what you measure: a systematic literature review examining whether young people in poorer socioeconomic circumstances are more at risk of chlamydia. *Sex Transm Infect* 2013;89(2):175-80. doi: 10.1136/sextrans-2011-050223 [published Online First: 13 October 2012]
22. Office for National Statistics. Standard Occupational Classification 2010: volumes 1-3. Basingstoke: Palgrave Macmillan, 2010.
23. Bailey JA, Haggerty KP, White HR, et al. Associations between changing developmental contexts and risky sexual behavior in the two years following high school. *Arch Sex Behav* 2011;40(5):951-60. doi: 10.1007/s10508-010-9633-0 [published Online First: 23 June 2010]
24. Schwartz SJ, Zamboanga BL, Ravert RD, et al. Perceived parental relationships and health-risk behaviors in college-attending emerging adults. *J Marriage Fam* 2009;71(3):727-40. doi: 10.1111/j.1741-3737.2009.00629.x [published Online First: 30 July 2009]
25. Lyons H, Manning W, Giordano P, et al. Predictors of heterosexual casual sex among young adults. *Arch Sex Behav* 2013;42(4):585-93. doi: 10.1007/s10508-012-0051-3 [published Online First: 08 January 2013]
26. Chanakira E, O'Cathain A, Goyder EC, et al. Factors perceived to influence risky sexual behaviours among university students in the United Kingdom: a qualitative telephone interview study. *BMC Public Health* 2014;14:1055. doi: 10.1186/1471-2458-14-1055 [published Online First: 09 October 2014]
27. Padilla-Walker LM, Nelson LJ, Madsen SD, et al. The role of perceived parental knowledge on emerging adults' risk behaviors. *J Youth Adolesc* 2008;37(7):847-59. doi: 10.1007/s10964-007-9268-1 [published Online First: 02 February 2008]
28. El Ansari W, Sebena R, Stock C. Socio-demographic correlates of six indicators of alcohol consumption: survey findings of students across seven universities in England, Wales and Northern Ireland. *Arch Public Health* 2013;71:29. doi: 10.1186/2049-3258-71-29 [published Online First: 06 November 2013]
29. El Ansari W, Vallentin-Holbech L, Stock C. Predictors of illicit drug/s use among university students in Northern Ireland, Wales and England. *Glob J Health Sci* 2014;7(4):18-29. doi: 10.5539/gjhs.v7n4p18 [published Online First: 16 December 2014]
30. IOM (Institute of Medicine) and NRC (National Research Council). The Science of Adolescent Risk-Taking: Workshop Report. Washington, DC: Committee on the Science of Adolescence, 2011.
31. Bynner J. British youth transitions in comparative perspective. *J Youth Stud* 2001;4(1):5-23. doi: 10.1080/13676260120028529
32. Fenton KA, Mercer CH, McManus S, et al. Ethnic variations in sexual behaviour in Great Britain and risk of sexually transmitted infections: a probability survey. *Lancet* 2005;365(9466):1246-55. doi: 10.1016/S0140-6736(05)74813-3 [published Online First: 07 April 2005]
33. Finney N. Understanding ethnic differences in the migration of young adults within Britain from a lifecourse perspective. *Transactions of the Institute of British Geographers* 2011;36(3):455-70. doi: 10.1111/j.1475-5661.2011.00426.x [published Online First: 17 March 2011]
34. Wayal S, Hughes G, Sonnenberg P, et al. Ethnic variations in sexual behaviours and sexual health markers: findings from the third British National Survey of Sexual Attitudes and Lifestyles (Natsal-3). *Lancet Public Health* 2017;2(10):e458-e72. doi: 10.1016/S2468-2667(17)30159-7 [published Online First: 24 October 2017]

For peer review only - http://bmjopen.bmj.com/site/about/guidelines.xhtml

35. Copas AJ, Wellings K, Erens B, et al. The accuracy of reported sensitive sexual behaviour in Britain: exploring the extent of change 1990–2000. *Sex Transm Infect* 2002;78:26-30. doi: 10.1136/sti.78.1.26
36. Prah P, Copas AJ, Mercer CH, et al. Consistency in reporting sensitive sexual behaviours in Britain: change in reporting bias in the second and third National Surveys of Sexual Attitudes and Lifestyles (Natsal-2 and Natsal-3). *Sex Transm Infect* 2014;90(2):90-3. doi: 10.1136/sextrans-2013-051360 [published Online First: 25 November 2013]

For peer review only

For peer review only - http://bmjopen.bmj.com/site/about/guidelines.xhtml

Figure 1 legend

Household structure by gender and age-group among the resident British population aged 16-74 years.

For peer review only

For peer review only - http://bmjopen.bmj.com/site/about/guidelines.xhtml

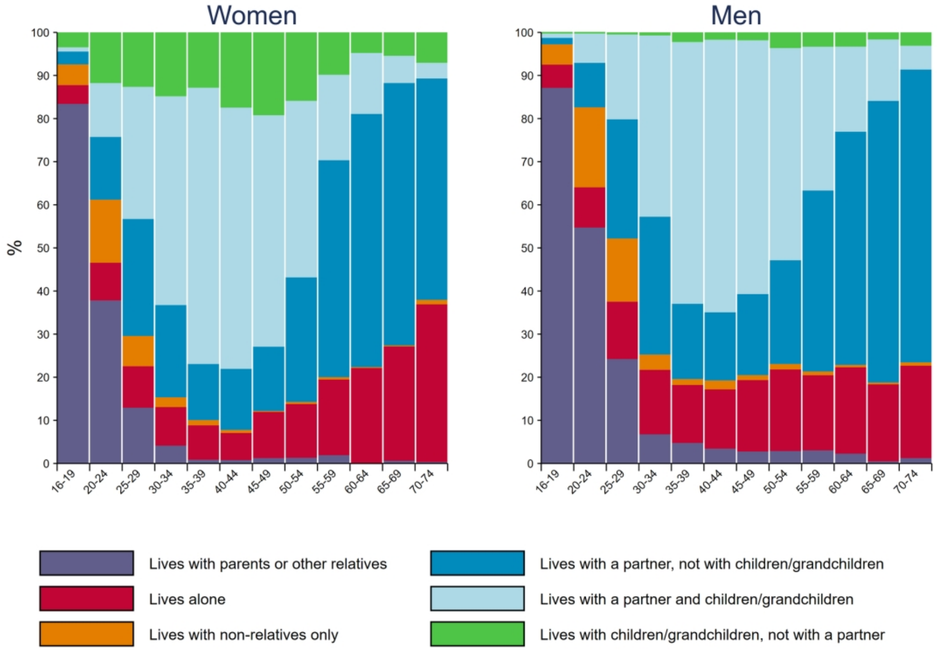

Figure 1: Household structure by gender and age-group among the resident British population aged 16-74 years

180x130mm (300 x 300 DPI)

STROBE (Strengthening The Reporting of OBservational Studies in Epidemiology) Checklist

A checklist of items that should be included in reports of observational studies. You must report the page number in your manuscript where you consider each of the items listed in this checklist. If you have not included this information, either revise your manuscript accordingly before submitting or note N/A.

Note: An Explanation and Elaboration article discusses each checklist item and gives methodological background and published examples of transparent reporting. The STROBE checklist is best used in conjunction with this article (freely available on the Web sites of PLoS Medicine at http://www.plosmedicine.org/, Annals of Internal Medicine at http://www.annals.org/, and Epidemiology at http://www.epidem.com/). Information on the STROBE Initiative is available at www.strobe-statement.org.

Section and Item	Item No.	Recommendation	Reported on Page No.
**Title and Abstract**	1	(*a*) Indicate the study's design with a commonly used term in the title or the abstract	
		(*b*) Provide in the abstract an informative and balanced summary of what was done and what was found	
**Introduction**			
Background/Rationale	2	Explain the scientific background and rationale for the investigation being reported	
Objectives	3	State specific objectives, including any prespecified hypotheses	
**Methods**			
Study Design	4	Present key elements of study design early in the paper	
Setting	5	Describe the setting, locations, and relevant dates, including periods of recruitment, exposure, follow-up, and data collection	
Participants	6	(*a*) *Cohort study*—Give the eligibility criteria, and the sources and methods of selection of participants. Describe methods of follow-up<br><br>*Case-control study*—Give the eligibility criteria, and the sources and methods of case ascertainment and control selection. Give the rationale for the choice of cases and controls<br><br>*Cross-sectional study*—Give the eligibility criteria, and the sources and methods of selection of participants	
		(*b*) *Cohort study*—For matched studies, give matching criteria and number of exposed and unexposed<br><br>*Case-control study*—For matched studies, give matching criteria and the number of controls per case	
Variables	7	Clearly define all outcomes, exposures, predictors, potential confounders, and effect modifiers. Give diagnostic criteria, if applicable	

For peer review only

Section and Item	Item No.	Recommendation	Reported on Page No.
Data Sources/ Measurement	8*	For each variable of interest, give sources of data and details of methods of assessment (measurement). Describe comparability of assessment methods if there is more than one group	
Bias	9	Describe any efforts to address potential sources of bias	
Study Size	10	Explain how the study size was arrived at	
Quantitative Variables	11	Explain how quantitative variables were handled in the analyses. If applicable, describe which groupings were chosen and why	
Statistical Methods	12	(*a*) Describe all statistical methods, including those used to control for confounding	
		(*b*) Describe any methods used to examine subgroups and interactions	
		(*c*) Explain how missing data were addressed	
		(*d*) *Cohort study*—If applicable, explain how loss to follow-up was addressed<br>*Case-control study*—If applicable, explain how matching of cases and controls was addressed<br>*Cross-sectional study*—If applicable, describe analytical methods taking account of sampling strategy	
		(*e*) Describe any sensitivity analyses	
**Results**			
Participants	13*	(a) Report numbers of individuals at each stage of study—eg numbers potentially eligible, examined for eligibility, confirmed eligible, included in the study, completing follow-up, and analysed	
		(b) Give reasons for non-participation at each stage	
		(c) Consider use of a flow diagram	
Descriptive Data	14*	(a) Give characteristics of study participants (eg demographic, clinical, social) and information on exposures and potential confounders	
		(b) Indicate number of participants with missing data for each variable of interest	
		(c) *Cohort study*—Summarise follow-up time (eg, average and total amount)	
Outcome Data	15*	*Cohort study*—Report numbers of outcome events or summary measures over time	
		Case-control study—Report numbers in each exposure category, or summary measures of exposure	
		Cross-sectional study—Report numbers of outcome events or summary measures	

For peer review only

Section and Item	Item No.	Recommendation	Reported on Page No.
Main Results	16	(*a*) Give unadjusted estimates and, if applicable, confounder-adjusted estimates and their precision (eg, 95% confidence interval). Make clear which confounders were adjusted for and why they were included	
		(*b*) Report category boundaries when continuous variables were categorized	
		(*c*) If relevant, consider translating estimates of relative risk into absolute risk for a meaningful time period	
Other Analyses	17	Report other analyses done—eg analyses of subgroups and interactions, and sensitivity analyses	
**Discussion**			
Key Results	18	Summarise key results with reference to study objectives	
Limitations	19	Discuss limitations of the study, taking into account sources of potential bias or imprecision. Discuss both direction and magnitude of any potential bias	
Interpretation	20	Give a cautious overall interpretation of results considering objectives, limitations, multiplicity of analyses, results from similar studies, and other relevant evidence	
Generalisability	21	Discuss the generalisability (external validity) of the study results	
**Other Information**			
Funding	22	Give the source of funding and the role of the funders for the present study and, if applicable, for the original study on which the present article is based	

*Give information separately for cases and controls in case-control studies and, if applicable, for exposed and unexposed groups in cohort and cross-sectional studies.

Once you have completed this checklist, please save a copy and upload it as part of your submission. DO NOT include this checklist as part of the main manuscript document. It must be uploaded as a separate file.

For peer review only
